# Part I: understanding pain in pigs—basic knowledge about pain assessment, measures and therapy

**DOI:** 10.1186/s40813-025-00421-0

**Published:** 2025-03-11

**Authors:** Julia Kschonek, Lara Twele, Kathrin Deters, Moana Miller, Jennifer Reinmold, Ilka Emmerich, Isabel Hennig-Pauka, Nicole Kemper, Lothar Kreienbrock, Michael Wendt, Sabine Kästner, Elisabeth grosse Beilage

**Affiliations:** 1 Institute for Biometry, Epidemiology and Information Processing (IBEI), University of Veterinary Medicine, Foundation, Hannover, Bünteweg 2, 30559 Hannover, Germany; 2 Clinic for Horses, University of Veterinary Medicine, Foundation, Hannover, Bünteweg 9, 30559 Hannover, Germany; 3 Field Station for Epidemiology, University of Veterinary Medicine, Foundation, Hannover, Büscheler Str. 9, 49456 Bakum, Germany; 4 Institute for Animal Hygiene, Animal Welfare and Farm Animal Behavior, University of Veterinary Medicine, Foundation, Hannover, Bischofsholer Damm 15, 30173 Hannover, Germany; 5 Institute of Pharmacology, Pharmacy and Toxicology, Faculty of Veterinary Medicine, University Leipzig, An den Tierkliniken 39, 04103 Leipzig, Germany; 6 Clinic for Swine and Small Ruminants, Forensic Medicine and Ambulatory Service, University of Veterinary Medicine, Foundation, Hannover, Bischofsholer Damm 15, 30173 Hannover, Germany; 7 Clinic for Small Animals, University of Veterinary Medicine, Foundation, Hannover, Bünteweg 2, 30559 Hannover, Germany

**Keywords:** Nociception, Inflammatory pain, Neuropathic pain, Clinical pain, Pain parameters, Pain therapy

## Abstract

**Background:**

Pigs can suffer from pain due to spontaneously occurring diseases, wounds, injuries, trauma, and physiological conditions such as the farrowing process; however, this pain is often neglected. To increase knowledge and awareness about this phenomenon, the current article presents a scoping review of basic and new approaches for identifying, evaluating, and treating pain in pigs.

**Methods:**

A scoping review was conducted with results from a search of the electronic database VetSearch and CABI. With regard to eligibility criteria, 49 out of 725 publications between 2015 and the end of March 2023 were included. The findings are narratively synthesized and reported orienting on the PRISMA ScR guideline.

**Results:**

The results of this review showed that practitioners need to consider pain not only as a sign of a disease but also as a critical aspect of welfare. If both the symptoms of pain and the underlying reasons remain unassessed, the longevity and prosperity of pigs may be at risk. In this respect, veterinarians are obliged to know about intricacies of pain and pain mechanisms and to provide adequate treatment for their patients.

**Conclusion:**

It is pivotal to increase knowledge about pain mechanisms, the reasons for heterogeneity in behavioural signs of pain, and methods for evaluating whether a pig is experiencing pain. This article will help practitioners update their knowledge of this topic and discuss the implications for everyday practice.

**Supplementary Information:**

The online version contains supplementary material available at 10.1186/s40813-025-00421-0.

## Introduction

Untreated pain in animals is associated with suffering, distress and detrimental effects on physical and mental health and thus represents a welfare-related concern [1]. The causes and indicators of pain are less examined in pigs than in companion animals [2], and pain management in pigs is often disregarded in textbooks [3]. Indeed, pigs are still among the most neglected livestock species in terms of pain assessment and treatment [4]. Although some related studies have been published, the focus is often limited to certain topics. For example, publications examining pain assessment have focused on pain management procedures (surgical castration, tail docking, teeth grinding, ear tagging or notching). Other studies have focused on pigs that are used as laboratory animals in translational medicine [5–11]. This is likely due to the critical discussion on the necessity of husbandry and laboratory procedures. However, despite being a serious welfare concern, pain caused by spontaneously occurring diseases or injuries including, wounds, trauma and physiological conditions like neuroma among pigs has been less well examined and reported [3, 11, 12]. The reason for this difference may be that pain directly induced by human intervention gains more attention than pain resulting from spontaneously occurring diseases or injuries. Another reason may be that veterinarians need to learn giving more consideration to pain management, as shown by a survey of veterinarians’ use of analgesics in livestock animals [13]. In general, it is the responsibility of a veterinarian to try to successfully alleviate pain in the animals under care [2]; however, achieving this goal can be complicated by difficulties associated with identifying pain. The identification and grading of pain needs to be a necessary part of clinical examinations of individual pigs. However, clinical examinations often focus on aetiological diagnoses, while the role or presence of disease-related pain is not always of concern. Consequently, therapy often aims to resolve the cause of the disease and neglects to treat the related pain.

For several reasons, it is important for a veterinary practitioner to be able to identify pain as an important symptom in pigs, thus enabling the veterinarians to choose an appropriate therapy and to monitor the effectiveness of the therapy. In the role of an advisor, for example, a veterinarian must support farmers in discharging their responsibility to protect their pigs from unnecessary pain and suffering [14]. Moreover, severe pain, which cannot be effectively treated, is a common reason for euthanasia or emergency killing of a pig in practice. In this respect, thoroughly assessing the animal for the presence of possible pain states ensure that the correct approach is selected in jurisdictional terms, where emergency killing is defined as “[…] the killing of animals which are injured or have a disease associated with severe pain or suffering and where there is no other practical possibility to alleviate this pain or suffering” [15].

In summary, the identification and evaluation of pain in pigs is pivotal for ensuring the welfare and prosperity of pigs and for deciding about timely euthanasia in severe cases. To support these pivotal processes, this article summarizes the knowledge and understanding of pain and related mechanisms. This article is a starting point for readers to become familiar with pain research and pain in pigs (Part I). Moreover, findings from the latest publications are presented to suggest how daily practice can benefit from findings in research. Building upon this review, another article addresses the state of knowledge on pain in specific, spontaneously occurring diseases and injuries in pigs (Part II).

## Method for the review

The aim of this scoping review is to enhance the understanding of pain and related mechanisms in pigs. In addition to summarizing the basic literature on the subject, topics and new approaches to assess pain in pigs were examined in studies published between 2015 and the end of March 2023. This scoping review was conducted in accordance with the PRISMA-ScR reporting guideline [16].

The search database “VetSearch” (EBSCOhost Research Database) was used which includes the following databases: CAB Abstracts 1990-Present, Tierärztliche Hochschule Hannover Catalogue, CAB Abstracts, CAB Abstracts Archive, eBook Collection (EBSCOhost), ERIC, E-Journals, OpenDissertations, MEDLINE, and Global Health. Thus, studies from key publishers (such as Wiley, Springer, Wiley-Blackwell, Taylor & Francis, Elsevier, and MDPI Biomedical Central Ltd., Cambridge University Press, among others) were included and addressed with the help of one single interface (one search mask). To control the search process and adhere to the journal requirements, we iterated the research steps in the Cabi Rxiv database in the English language.

To find appropriate publications, two alternate search strings were used. Findings for the first search string are called version 1 (V1) for results of the search in VetSearch and version 3 (V3) for CABI Rxiv. Findings for the second search string are called version 2 (V2) for results of the search in VetSearch and version 4 (V4) for CABI Rxiv. The following search terms were used: (“pain”) in title (V1, V3) or keywords (V2, V4) AND in text ("pig" OR "pigs" OR "hog" OR "hogs" OR "porcine" OR "swine" OR "boars" OR "boar" OR "sow" OR "sows" OR "piglet" OR "piglets" OR "weaner" OR "weaners") AND in text ("nocicept*" OR "hurt*" OR "suffer*" OR "damag*" OR in text "injur*" OR "defect*" OR "harm" OR "sensation" OR "burden" OR "sensorium") AND NOT in text (“patients”).

In brief, 715 publications were found. For the first screening step, the list of retrieved publications was assessed online (title, author, abstract) or downloaded and an overview of topics was generated, (the topics correspond to the chapters of the manuscript nociception, inflammation, therapy (non-husbandry interventions; husbandry interventions), neuropathic pain and assessment, other animals or topics). Papers were considered eligible if they were peer reviewed, accessible in either article or book (section) format and published between 2015 and March 2023. In the second screening step, a more detailed analysis was performed, and papers were assessed for the fit of addressing the principles of pain in pigs and pain in spontaneously occurring diseases and injuries. Papers were considered eligible after this step if they addressed one of the respective topics and presented results or reviews of clinical studies. Accordingly, papers were excluded if they focused too much on pain management procedures (e.g., docking, castration, ear notching) or if “pain” or related concepts were only addressed as a buzzword. Moreover, papers were excluded if they elaborated mainly on the discourse, ethics or attitudes of people concerning the pain of pigs. In cases where no publication was found, papers were retrieved following a snowballing technique. As outlined before, commonly used papers, standard books and literature published before 2015 were also integrated. By help of this iterative screening process, 49 publications were collected for the search and review process. Additional metrics of the search can be seen in supplementary materials (Additional file 1). For reporting, the most suitable paper was selected as the lead reference if several papers addressed the same aspect. To illustrate particular sections, additional material is provided as pictures and video footage. The material is based on a study elaborating on timely euthanasia of pigs suffering from pain and distress on German farms [17].

### Definitions and (patho-)physiology of pain

Research on pain has been conducted for centuries, and the definition of pain has evolved over time. In the following, the most relevant definitions and perspectives on pain and pain mechanisms are presented together with a narrative report of findings from the review.

### Pain and nociception

The *International Association for the Study of Pain* (IASP) is often cited both in human and veterinary medicine as the first reference to provide a definition of pain. Its latest and adapted version outlines that pain is “[a]n unpleasant sensory and emotional experience associated with, or resembling that associated with, actual or potential tissue damage.” ([18], Text Box 2). While the IASP explicitly addresses the pain experience of animals now, earlier definitions emphasized the focus on the animals’ response to pain: … [pain] changes the animal’s physiology and behaviour to reduce or avoid the damage, to reduce the likelihood of recurrence and to promote recovery…” ([19], p.266). A key aspect to bear in mind, in this respect, is that the inability to communicate the pain experience verbally does not negate the possibility that an individual is experiencing pain and requires appropriate pain relief (cf. [18, 20, 21]). Because humans and vertebrates share similar neuroanatomical structures associated with pain processing, painful events in humans are also very likely to occur in vertebrates [22]. In fact, the principle of analogy is often used to justify the use of animals, including pigs, in the study of human pain or to argue for considering pain in painful conditions [1, 5, 11, 12]. To date, numerous studies have proven this assumption and outlined a great set of shared physiological pain mechanisms, especially for pigs [23, 24].

Moving from the definition of pain to the topic of ‘pain mechanisms’, however, requires defining the term nociception. Nociception describes the reception of stimuli by nerve cell endings, called nociceptors. It comprises a process by which the body encodes potentially or actually damaging stimuli and initiates a series of events required to transmit that information to the brain [25, 26]. Hence, the activation of nociceptors themselves does not necessarily result in pain [25, 27]. In contrast, pain perception arises through cortical processing and comprises emotional and perceptual (conscious) experiences [25]. In other words, nociception may not always lead to pain and other types of pain may occur without nociception (see an overview in Table 1).

**Table 1:** Types of pain, including the description and biological function [28]

Pain type		Nociceptive pain	Inflammatory pain	Neuropathic pain
Description		Pain caused by physiological activation of peripheral high threshold nociceptors *(subcategories include somatic and visceral pain)*	Spontaneous and stimulus- dependent pain evoked by both low-and high-intensity stimuli	Spontaneous pain caused by lesions or disease of the somatosensory nervous system
Biological function	Adaptive (physiological)	Protection of the organism from injury	Protection by hypersensitivity during healing and repair	–
	Maladaptive (patho-physiological)	Persistent pain despite healing and absence of initial causes or triggers, may become a disease entity** on its own		No protective function; may become a disease entity** on its own
References from the review*		Pig as a model [24, 29] Pigs in the focus of the publication [30–36]	Pig as model [37–40] Pigs in the focus of the publication [41, 42]	Pig as a model [43, 44] Pigs in the focus of the publication [30, 31, 45, 46]

*Overview, some studies address several aspects; ** see [47]

Types of pain are characterized by the different mechanisms causing it (Table 1), but in clinical pain, they overlap and evolve over time. To facilitate reading, the following paragraphs are structured according to Table 1, as is common in the standard literature.

### Nociceptive pain

#### Processes of nociception

**Fig. 1:**
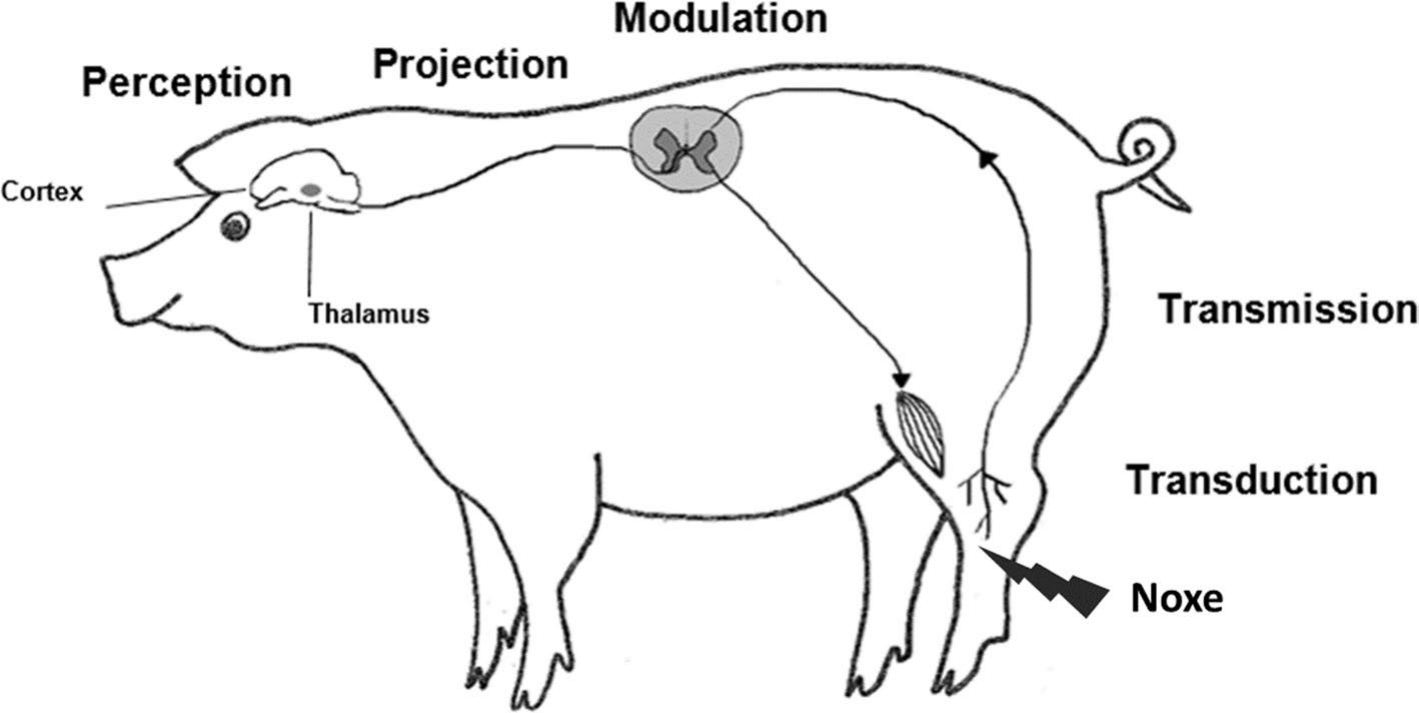
Schematic diagram of physiological nociceptive pathways by E. grosse Beilage (oriented on [3, 21])

Nociceptive pain is caused by the physiological activation of peripheral high-threshold nociceptors. It plays an important role in the protection of the body from further injury by initiating reflex and avoidance responses [25]. Nociceptive pain can be induced by polymodal, peripheral sensory neurons (nociceptors) responding to noxious thermal, mechanical, or chemical stimuli. Nociceptors encode the quality and quantity (e.g., duration, intensity, location) of noxious stimuli and transduce them into depolarizing action potentials (*transduction*). Nociceptive impulses are then transmitted to the spinal cord by specialized afferent nociceptor fibres and Aδ- and C-fibres (transmission), [24, 27, 48]. The afferent nerve fibres enter the dorsal horn of the spinal cord. At that point, signal inhibition or amplification (modulation) occurs before the information is conveyed to the brainstem, thalamus, limbic system and cortex (projection). Finally, complex processing of sensory nociceptive signals can result in the perception of pain [26]. These processes are illustrated in Fig. 1.

Importantly, the transmission of nociceptive pain must not be understood as rigid. It is subject to plasticity since modulation is a complex molecular process occurring at different levels of the central nervous system [49, 50]. Moreover, individual experience and factors, such as the type of initial fibre conduction, influence pain sensation. The initial pain, for example, is mediated by activation of thinly myelinated, fast-conducting Aδ fibres and can be perceived as brief, pricking and well-localized sensations eliciting protective responses (e.g., immediate motor withdrawal response). The subsequent pain is mediated by unmyelinated, slow-conducting C-fibres that account for long-lasting, burning and less well-localized pain [51, 52]. The second pain seems to initiate (long-term) behavioural responses to limit further injury [52].

#### Anatomical location of nociceptive pain

The anatomical location of tissue damage is associated with several typical characteristics, such as the experience and expression of nociceptive pain. In this respect, nociceptive pain can be differentiated into superficial and deep somatic pain (skin, subcutis, muscles, joints, bones) and visceral pain (organs of the thoracic, abdominal, or pelvic cavities). Superficial somatic pain is initiated by the activation of nociceptors in the skin and mucous membranes, which are highly innervated. Therefore, this type of pain is well localized. Deep somatic pain originates from bones, muscles, joints, and connective tissues and is less well localized. Visceral pain originates from distension of hollow organs, mesenteric traction, ischaemia, and endogenous inflammatory mediators [26]. It possesses exclusive characteristics concerning perception and perceived anatomical location: the liver, lung and kidney parenchyma, for example, are insensitive to pain, while the capsule of the liver and kidney and the parietal pleura possess nociceptors [53]. Visceral injury does not necessarily result in visceral pain (e.g., cutting the intestine), while distention or traction may cause pain without injuring the tissues [54]. Pain due to infections of the viscera, such as gastrointestinal disease, is commonly judged to be very painful for pigs [12, 55]. Hence, diagnosing the source of visceral pain might be challenging because the underlying pathology and the intensity of pain perceived by the individual animal are not necessarily closely correlated [26]. Moreover, visceral pain is diffuse and poorly localized due to the sparse innervation of visceral organs and the spread of visceral afferents across several laminae as well as segments when terminating in the spinal cord, thereby inducing large receptive fields [49, 54]. Due to convergence of visceral and somatic nociceptive input in the spinal cord, visceral pain is often characterized as referred pain, meaning that pain is perceived adjacent to or at a distance from the noxious stimulus, typically at somatic sites (e.g., angina pectoris in humans leading to pain in the arm) [49, 54]. Finally, visceral pain can be accompanied by emotional (affective) and autonomic responses such as nausea, vomiting, sweating and changes in blood pressure and heart rate because of autonomic innervation of the visceral organs [53, 54].

#### Duration of nociceptive pain

The sensation of pain can be further differentiated into acute and chronic pain, depending on how long the sensation lasts. Acute pain (or adaptive pain) has a protective function and is essential to the organism because it enables healing and tissue repair and thus the animal’s wellbeing [56]. Chronic pain was arbitrarily defined as pain persisting or recurring for more than 3 months [57]. In addition to the time span, initiated alterations in pain pathways and induced changes in the nervous system are of particular concern. The latter may contribute to physiologic, metabolic, and immunologic alterations [25] and affect the quality of life of animals [58, 59]. Hence, chronic pain refers to maladaptive or pathological pain that has no protective effect and should not be regarded as a continuation of acute pain [60].

### Inflammatory pain

Inflammation is a physiological response of the body to noxious stimuli, including (surgical) trauma or infection [56, 61], that is intended to evoke protective behaviour to encourage healing. Inflammatory pain often accompanies diseases and injuries and is accompanied by a set of well-defined pathophysiological characteristics. Therefore, it is important to understand more about its nature. A variety of proinflammatory agents and mediators (e.g., H^+^, prostaglandins, bradykinin, cytokines, nerve growth factor) are liberated following insult (see also paragraph biomarkers) and sensitize nociceptive fibres directly or indirectly [62, 63].

Stimulation of nociceptors also leads to reverse (antidromic) activation of C-fibres and subsequent release of neuropeptides, notably substance P (SP) and calcitonin gene-related peptide (CGRP). These peptides induce vasodilation, plasma extravasation, oedema, and further sensitization of nociceptors and thus contribute to neurogenic inflammation [64, 65]. It is well known that complex bidirectional neuroimmune interactions modulate inflammation and pain [66]. In this context, nerve growth factor (NGF) was found to be an important signalling molecule involved in mediating postoperative and osteoarthritic (OA) pain. Briefly, its interaction with the tropomyosin receptor kinase A receptor (TrkA) has been demonstrated to induce alterations in primary afferent nerve fibres and immune cells, sustaining and enhancing pronociceptive states [67]. Recently, anti-NGF monoclonal antibodies have been approved for the treatment of osteoarthritic pain in dogs [68] and cats [69].

Most of the literature included in this review assessed inflammatory pain in pigs, with translational interest in inflammatory skin diseases in general [38]. Practitioners can refer to these and other findings about cutaneous hyperalgesia (i.e., abnormally increased sensitivity to pain in response to a normally painful stimulus) due to UV-B irradiation [37, 40] when examining an individual pig with impaired skin conditions or sunburn and the need to judge upon the pain state. While the depth of findings cannot be resumed at this location, behaviour appears to be a valid parameter for observing inflammatory pain and hyperalgesia following irradiation, at least in familiar or controlled environments [37].

### Neuropathic pain

Neuropathic pain is initiated by lesions of the somatosensory system [20, 28]. This pain may result from peripheral or central nerve injury following acute events (e.g., amputation, spinal cord injury, freezing) or systemic or local diseases (e.g., viral infection, neoplasia) [26, 61]. Following such damage, a cascade of neurochemical and neuroplastic changes and altered expression of ion channels can lead to spontaneous painful sensations without an associated stimulus. Unlike inflammatory pain, which often subsides after the stimulus is eliminated, neuropathic pain can persist or become chronic [22]. Neuropathic pain can therefore be regarded as a maladaptive phenomenon leading to severe and long-term consequences for quality of life in humans [70] and animals [3].

### Sensitization and altered pain states

In addition to the protective function of nociceptive pain, high-intensity and/or prolonged noxious stimuli can result in sensitization [26]. Sensitization of the nociceptive system can be longer lasting but is reversible and evokes protective processes to avoid further injury [71]. As described above, tissue injury and inflammation liberate a variety of mediators (‘sensitizing soup’) [64], creating an altered molecular environment that leads to a reduction in the activation threshold and an increase in the responsiveness of peripheral nociceptors [26, 64, 72]. This so-called peripheral sensitization is closely linked to the site of tissue damage [73].

Intense, prolonged or repeated nociceptor input can trigger the excitability and pain transmission of neurons in central nociceptive pathways (i.e., the spinal cord and supraspinal structures) [22, 74]. Additionally, a reduction in inhibitory pathways and the recruitment of subthreshold synaptic inputs may lead to increased action potential output [71]. These processes of pain facilitation and pain disinhibition may contribute to a state called central sensitization. Consequently, central sensitization to nociceptive and innocuous stimuli is characterized by diffuse pain sensitivity and pain hypersensitivity. In contrast to peripheral sensitization, central sensitization is subject to changes in the properties of neurons in the central nervous system, meaning that painful sensations occur even after a stimulus is withdrawn [71]. Moreover, inputs to dorsal horn neurons from the activation of low-threshold Aβ fibres, which normally convey innocuous tactile stimuli, may contribute to central sensitization [75]. All these phenomena emphasize the plasticity of the somatosensory nervous system in response to activity, inflammation, and neural injury [71]. It should be noted here that neuropathic pain and central sensitization are not synonymous since the latter is initiated by intense or prolonged nociceptive inputs, irrespective of the origin of pain (nociceptive, inflammatory, or even neuropathic) [72].

Sharing some characteristics of central sensitization is the temporal summation of pain caused by repeated C-fibre stimulation or ‘wind-up’. It describes an increased pain sensation that is caused by repeated noxious stimuli [26]. Some of the mechanisms of ‘wind-up’ are thought to be related to altered pain states [76]. Molecular factors that contribute to central sensitization include N-methyl-D-aspartate receptor (NMDA)-mediated signalling, disinhibition, and microglial activation, among others [56, 62]. Overall, peripheral and central sensitization may contribute to altered pain states such as hyperalgesia (i.e., an exaggerated and prolonged response to noxious stimuli) and allodynia, a condition in which pain is caused by an innocuous stimulus (e.g., touching the skin) [22, 71].

### Clinical pain

The above-mentioned categorization of pain types provides a good overview and understanding of the complex topic of “pain”. Nonetheless, the clinical pain that practitioners encounter on a daily basis is usually a mixture of different pain types. This can be illustrated using the example of tail biting [77]. Following the initial insult, acute superficial somatic pain may be suspected. Over the course of time, due to the necrotizing purulent character of those lesions, inflammatory pain and possibly neurogenic inflammatory processes emerge. Depending on the degree of neural injury, neuropathic pain is likely to develop. Indeed, in an experimental study of pigs that underwent tail amputations, sensitization and sustained alterations in peripheral sensitivity resembling neuropathic pain were observed [31]. In fact, the transition from physiological to pathological pain conditions often occurs frequently. Pathological or maladaptive pain has no protective function [64]. This pain state is mostly persistent or recurring, even long after the traumatic event or illness subsides or if acute pain is inappropriately managed or untreated.

This latter factor is especially important for practitioners. Even if the initial cause is absent, pain due to traumatic lesions may (re)occur over time. Initial ideas on how to assess and validate pain in amputated body parts in this regard may be inferred by studies elaborating on tail amputations [30, 31, 45, 46, see chapter nociception]. Another example is for practitioners who face chronically lame animals. Indicators such as the walking pattern (among others), as well as hints for diagnostic anaesthesia and evaluation protocols, may be derived from studies on neuropathic pain models [43, 44].

### Pain as a disease entity

Although one incident can activate several pain types, the sensation of pain may also appear or be sustained irrespective of the trigger, cause or healing process. Pain may be a self-standing disease entity in this respect, and pigs should be examined and treated for this diagnosis, similar to any other common swine disease.

Pain as a disease entity in pigs includes both acute and chronic pain. Although it is difficult to diagnose pain in pigs (and treat different pain types with respect to available medication), the consequences in terms of welfare and costs of neglected cases are high [58, 59]. If no pain alleviation is possible, pigs may even have to be euthanized with respect to the definition of mercy killing [15].

In human medicine, discussions on how to diagnose and define pain as a disease entity are currently underway [47, 78]. Future studies on pain in pigs should elaborate on this topic as well, but in the first step, awareness of the need to document the diagnosis of pain as well as the appropriate treatment needs to be improved.

### Implications and outlook

What further implications do the terms and definitions of pain and pain mechanisms have for everyday practice? One answer is that practitioners can apply the updated knowledge and re-evaluate individual cases. For example, when examining a pig with accidently amputated body parts such as a dew claw, a veterinarian should determine whether common signs of chronic pain appear, as described in recent republications [30, 31, 45, 46, 79]. Another answer is that the updated knowledge leads to a change in perspective: rather than assuming that pain is not present in a pig, veterinarians should ask if enough evidence is present to reject the assumption that an individual pig is experiencing pain. According to recent publications, individuals were asked whether a nonresponse to stimuli may be explained by the fact that the pig is distracted by examinations (cf. role of consciousness, [35]) or because it remembers previous routine visits and avoids being (painfully) re-examined (cf. role of habituation, [34, 36, 80]). Moreover, if a pig scores lower on pain scales than expected, practitioners should consider how this state was experienced by humans or whether the pig may simply belong to a type less expressive of pain, just as there are different personalities and coping styles among humans [59, 81].

In sum, incorporating the latest knowledge about terms and definitions of pain means that practitioners should focus on individual pigs and reconsider whether remote observation is needed or at least if additional time is needed to re-evaluate the first impression about individual pain states.

Furthermore, learning about the state of related research underlines how invaluable the perspective of pig veterinarians is for improving knowledge in the field. For example, few studies have examined pain due to gastrointestinal diseases and injuries [3, 82, 83] or urinary [84] and respiratory tract diseases [39]. Veterinarians who report field cases with the help of the above-defined terms will enhance the practice-research dialogue and refine the understanding of pain in pigs.

### Pain assessment in pigs

Assessing pain in pigs requires knowing well about the typical behaviour of the species as well as the potential idiosyncrasies of the individual since pigs often tend to hide their pain [2, 85, 86]. The indicators relevant for pigs range from physiological to behavioural aspects, and the latter is mostly used by practitioners [87]. Currently, no harmonized nomenclature or categorization of indicators has been established [3, 12]. While it is out of scope for this article to suggest a harmonized system, orienting to other fields shows that methods of pain assessment can differ according to the focus on spontaneous or evoked behaviour but also in terms of how the pain is scaled.

Using a “subjective verbal pain scale”, for example, a practitioner describes the pain state with qualifying words such as “moderate” or “severe” pain. Using a “categorical scoring systems”, these words were associated with numbers (mild, 1; moderate, 2; severe, 3), and a set of indicators was predefined for assessment (such as motion (movement behaviour, such as the movement to the feeder) or body condition (that can be affected by pain sensitivity). Once the scores are noted, they are weighted according to relevance for the species or disease to calculate the sum and thus overall pain score of the assessment. Several further pain scales exist (visual analogue, numerical rating, simple descriptive or grimace scales), and they were developed to improve the reliability, validity and objectivity of measuring pain in animals [21].

Concerning pain in pigs, similar efforts and discussions are underway [3, 12, 88]. For example, a recent study evaluated the value of behavioural pain scales for pigs. It was concluded that the overall evidence for the UPAPS (Unesp-Botucatu Pig Composite Acute Pain Scale) is strong and that the overall evidence for the PGS-B (Piglet Grimace Scale-B) is moderate for assessing pain in cases of castration and tail docking, respectively [88]. The use of these scales among practitioners who assess pain induced by spontaneously occurring diseases and injuries has yet to be assessed. However, these scales rely on indicators such as attention given to the affected area, interactive behaviour, ear position, and orbital tightening (spontaneous behaviour), which are also major concerns for stable veterinarians. Hence, knowing about recent developments in pain scales and indicators is essential for ensuring the best evaluations of pain in pigs.

For pigs unfamiliar with typical pain behaviour, however, the first step is to know and understand indicators before they can be detected in a pig. In this regard, the most common indicators of pain in pigs will be discussed and described with additional materials.

### Behavioural parameters

The term “behaviour” summarizes the overall sensomotoric expression of an animal [89] and is classified as abnormal when it differs in pattern, frequency or context from the behaviour shown by most members of the species [90]. Behavioural changes associated with pain have mainly been deduced from spontaneously occurring behaviours arising or increasing in the context of painful conditions induced by damaging management procedures [12]. Pain assessment based on behaviour analysis has the advantage that it is not invasive, does not require equipment or restraint, and can be assessed by remote observation [4]. Nonetheless, the evaluation of behaviour during a clinical examination might be confounded by pig-examiner interactions [12, 91].

Behaviour, evaluated in terms of pain, consists of the expression of various indicators, describing how a pig is reacting to its environment, interacting with pen mates, and showing vocalization, muscle activities and changes in posture or locomotion. A set of clinical parameters, such as the amount of time spent time walking, resting, sleeping, rooting, and interacting, as well as longer durations spent in an abnormal posture, walking with difficulty, and lying alone, have recently been validated to indicate pain after surgery [4]. Even though this fully validated scale for acute pain is based on longitudinal video analysis [4], it shows the general suitability of the parameters. To assess clinical signs of spontaneously occurring diseases and injuries, individuals of the same group or pen of unaffected pigs should serve as a reference during an on-farm examination of individuals [81, 82]. A comparison of this level and an evaluation of a set of indicators will help to identify substantial differences from normal behaviour [4], but subtle changes may be overlooked.

Several pain scales have been developed for various species and different purposes [4, 92–95], and as outlined in [88], pain scores in pigs are already under way. Future research could explore whether these are helpful tools for decision making about pain management in spontaneously occurring diseases or injuries or about pain treatment for veterinary practitioners. Moreover, it is important to consider and evaluate the behavioural changes known to be indicative of pain in pigs.

The parameter ‘attention’ summarizes how a pig responds to the environment, e.g., caretaker, examiner or noises from technical equipment. Unaffected pigs direct their attention towards any action. In affected pigs, reduced attention can range from listlessness (mild) to lethargic (severe) states [87]. However, restlessness can also indicate pain [96]. How pigs engage with their pen mates is called ‘social interaction’. Affected pigs exhibit self-separation [6, 87] by lying close to the wall or in corners and by reducing their encounters with other pigs. Indeed, social isolation may even be a more specific indicator of pain than general interaction behaviour [82]. Defence in dominance-related interactions with pen mates is reduced among pigs experiencing pain.

Reduced feed intake is one of the most frequently used overall indicators for disease but cannot be considered valid pain indicators, as specificity is likely low and difficult to evaluate in pigs fed ad libitum and housed in groups [87].

Interpretation of vocalization as a pain response requires consideration, as some painful events induce vocalization, while other pain-related events suppress vocalization [3]. There is clear evidence that vocalization is indicative of pain [3]. For example, technical analysis of individual vocalizations recorded from piglets showed that calls differed between various conditions (pain, cold and hunger) and could be detected with an accuracy rate of 81% [97]. In another study using multiparametric call analysis to classify vocalizations during castration pain, three call types were distinguishable (grunt, squeal, scream). In comparison, screams appeared to be pain related, as the piglets that were castrated without local anaesthesia produced almost twice as many screams as piglets that were castrated with anaesthesia. The screams during castration also became more extended and more powerful [98]. The total call energy, sound pressure level, peak-to peak pressure, maximum call frequency and temporal characteristics of the individual call can also be used as indicators of pain [99]. However, not every call represents pain; for example, inadequate handling may also provoke vocalization [100]. Hence, vocalizations must be examined in a particular context. For example, if a lame pig is screaming while walking without being moved forward, it is likely that the vocalization is an indicator of pain (Additional file 2).

Teeth grinding (bruxism) is also indicative of pain [87]. Identification of this characteristic noise under on-farm conditions requires an experienced examiner, as teeth grinding is often drowned out by other environmental noise (Additional file 3).

Tremor or trembling is a subtle indicator of pain. While shivering might be caused by low temperatures, tremors are limited to the skeletal muscles (Additional file 4) of a part of the body and are considered to indicate pain [12, 87, 101].

Tail posture can be used to indicate pain. Tail posture and motion are impaired in docked tails [102]. When tails are undocked or when only the tip is docked, a curled tail as well as a relaxed hanging or loosely wagging tail are associated with positive valence (emotional states) and high or low arousal, respectively. A constantly tucked, motionless tail indicates negative valence and low arousal, while a tail tucked in a sudden response to a threat is associated with negative valence and high arousal [102]. Tucked tails can be observed in cases of pain, sickness and fear. Tail tucking due to tail biting is often chronic and results in an almost permanently or frequently tucked tail [102, 103]. Tail wagging includes the side-to-side movement of the tail. Relaxed wagging (tail swinging) occurs during various social behaviours, locomotor and social play and locomotion. However, intense tail wagging in pigs with biting lesions can be a sign of distress, tail irritation, or pain and can occur in pigs that are victims of tail biting [102].

Body posture also provides valuable information about pain in pigs [3]. Kneeling is a strong indicator of pain and is aimed at relieving painful parts of the body, e.g., lower parts of the forelegs, hind legs or abdomen (Additional file 5 and Additional file 6). Tripping, i.e., the rapid change between burdening and not burdening a foot or leg, is also a response to pain and is indicative of pain in more than one foot or leg (Additional file 7). Standing motionless with the head down might be caused by pain but also indicates suffering [12]. Postural change while sitting is indicative of pain, e.g., when a pig is trying to unburden a hind leg by bending the spine to place the leg in an upper position (Additional file 8), pain may be reduced under pain treatment [42]. An arched spine while the pig is standing or moving is also a sign of pain located in the locomotor system or inner organs. Huddling, i.e., lying with at least three legs under the body or lying in a stiff position, is another sign of pain ([12], p.4).

Lameness is a reduction in weight borne and expressed by carrying a foot, favouring a leg, or being unable to get up and move and it is an important indicator of pain in terms of severity [12]. Lame pigs exhibit asymmetrical weight bearing between legs, increased step frequency or stand time, tip-toe walking and altered stride length [104–106]. A previous study stated that locomotor disorders do not necessarily result in pain [3], as individuals may be affected by a biomechanical abnormality [12]. In another publication, it was suggested that “[…] joint injuries may prevent normal movement of the joint, leading to stiffness in gait”, ([107], p.66) which may not be associated with pain. However, this statement may need to be interpreted with caution. In horses, osteoarthritis (OA) of the distal tarsal joints is a frequently diagnosed disorder causing lameness and is referred to as “bone spavin”. In general, medical and surgical treatments aim to accelerate fusion of the affected tarsal bones to provide pain relief [108]. However, even in horses undergoing surgical arthrodesis, the resolution of lameness took up to 12 months [108]. To the authors' knowledge, there is no comparative literature on pigs. However, anatomical confirmation of the corresponding joints differs between horses and pigs.

Grimace scales have been used to evaluate behavioural changes and the facial expressions of piglets, growers and sows induced by tissue damage or disease [109–113]. Facial expressions comprise a number of anatomically based actions, such as changes in the shape of the eyes, nose, cheeks, mouth and ears [114]. In piglets, orbital tightening might be an indicator of pain induced by castration [109, 115]. This phenomenon is evaluated in the PGS-B grimace scale (among ear position, cheek tightening, nose bulge), which has been shown to have a strong level of evidence [88]. However, the use of grimace scales for pain evaluation is not yet ready for use in practice, as it requires extensive video recording. Currently, research focusing on automated pain recognition based on deep learning models is of particular interest in numerous mammalian species [116–118]. Interestingly, a real-time facial recognition platform has been proposed for pigs and cows aiming to detect emotions [119]. In the future, we will show whether these systems have the potential to become reliable and valid tools in daily veterinary practice for detecting painful conditions.

### Physiological parameters (biomarkers)

In addition to behaviour, physiological parameters (Table 2) can indicate painful conditions in animals. Painful stimuli lead to activation of the sympathetic nervous system and release of catecholamines, resulting in physical reactions. These include changes in cardiopulmonary parameters, as well as changes in skin temperature, pale mucous membranes, mydriasis, salivation, and decreased activity in the gastrointestinal and urinary systems [3, 120]. As nociceptive indicators, cardiovascular parameters are often inconsistent and not pain specific, as they are influenced by many factors in addition to pain, such as stress; homeostatic mechanisms [121]; medications; and the intensity, type, and location of noxious stimuli [122]. Elevated catecholamine concentrations, as well as glucose and lactate concentrations resulting from catecholamine-stimulated glycogen mobilization [123], have been detected in porcine blood as indicators of pain, although mostly in association with damaging management procedures such as piglet castration and tail docking [3, 124].

**Table 2:** Physiological parameters and biomarkers

Category	Physiological parameter/biomarker	References
Activity of the autonomous nervous system	Heart rate/heart rate variability Arterial blood pressure Respiratory rate Temperature (rectal, skin, eye)	[127, 133–137]
Hormonal concentrations in blood, saliva or urine	*Adrenal axis:* ACTH, cortisol	[138, 139]
	*Sympathetic axis:* adrenaline, noradrenaline, chromogranin a	[140–142]
	Neuropeptides (substance p, beta-endorphin)	[143, 144]
Blood energetic metabolites	Glucose Free fatty acids Lactate	[82, 133, 139]
Blood concentrations of inflammatory markers^*^	Cytokines (interleukin-1, tumour necrosis factor-alpha) Acute phase proteins (serum amyloid a, c-reactive protein, haptoglobin, fibrinogen)	[42, 77, 143, 145, 146]
Enzymes	Creatine kinase Salivary α-amylase Total esterase activity and its components (lipase, cholinesterase etc.)	[127, 146, 147, 148]
Pterines	Neopterine Biopterine	[149, 150]
Proto-Oncogenes	c-Fos	[151, 152]

Physiological parameters and biomarkers that have been used for pain identification in pigs (adapted from [124] with exemplary studies for each category). * Inflammatory markers indicate the existence of an inflammatory state that may generate pain

Cortisol is the most commonly used blood parameter for assessing pain in pigs in experimental settings. Cortisol is a steroid hormone produced by the adrenal gland in response to stress, and it can also increase in response to pain [125]. Studies have widely examined cortisol/adrenocorticotropic hormone (ACTH) levels in blood plasma, serum and saliva in pigs in relation to pain [12]. Damaging management procedures aside, painful events such as intramuscular injections [126], lameness [127, 128], and rectal prolapse [127] were found to result in significantly elevated cortisol levels in blood or saliva. A more recent method of cortisol determination in pigs involves detection in bristles. Findings showed that, compared with those in control groups, pigs suffering from chronic pain from tail biting or lameness during their lifetime had elevated cortisol levels in bristles [129], while avoiding any invasive, painful procedures in piglets resulted in lower cortisol levels at weaning age [130]. Thus, cortisol levels in bristles could be a suitable indicator of animal welfare.

Additional biomarkers (included metabolic, immunological, and inflammatory markers) have been identified as indicators of pain in pigs [124]. Most of these biomarkers, however, were examined in experimental settings and have not been validated for pain assessment in individual pigs with naturally occurring diseases [3]. Overall, the determination of laboratory parameters has limited relevance for routine clinical pain assessment. These parameters do not always allow for a clear inference of pain, as some markers respond to both pain and stress [131] and show natural circadian variations in their concentration levels [132]. Moreover, the delay in diagnosis does not allow for rapid on-farm decisions, which are essential in cases of severe, spontaneous disease in individual pigs.

### Other approaches to measure pain

Although several indicators have been mentioned and even more may be discussed in other reviews [3, 12], forthcoming and digitally assessed approaches need more attention. Electroencephalography (EEG), for example, provides a summation of electrical activity arising from the cerebral cortex. Currently, the application of these methods is limited by the experimental setting [153–155].

Infrared thermography (IRT) is a technique used to evaluate inflammatory conditions in pigs [156]. It is commonly used in laboratory settings, but recent studies have used this technique under field conditions and found it to be very effective for the early identification and treatment of shoulder ulcers in sows [157].

Another digital device for assessing pain sensitivity in pig skin and underlying tissues (e.g., osteoarthritis, synovitis) is the hand-held pressure application measurement (PAM) device. This device enables the application of force and the monitoring/measurement of mechanical nociceptive thresholds. The approach is promising because of the need to constrain pigs during measurement, apply consistent stimuli (exertion of force per area) and gather objective and consistent evoked responses. [32, 34, 35, 158, 159].

In the future, practitioners may use and evaluate the role of cognitive tests (such as memory tests or spatial memory tasks) in the field. Especially for critics and as a complement to nociceptive measures, this approach will enhance the understanding of the affective-motivational dimension of pain (cf. [3, 12, 58, 160]).

More commonly used in current practice is analgesic treatment (diagnostic anaesthesia), which is a tool for identifying pain in an individual pig. The ability of analgesic drugs (or anaesthetic techniques) to alleviate the effects of tissue damage is indicative of the presence of pain [3, 114]. The response to an analgesic treatment allows, to a certain degree, us to conclude that pain, but not every medicine, is potent for all kinds of pain [161]. Hence, the absence of pain reduction after treatment may indicate that the chosen pathway for relief failed instead of assuming that pain was generally absent [162].

In summary, depending on the location and kind of injury or disease, practitioners can use and should combine a set of behavioural, physiological and digitally assisted approaches to elicit the pain state of an individual pig.

### Pain therapy in pigs

Veterinarians are responsible for providing the best possible treatment. As outlined in the previous paragraphs, there is no doubt that pigs can sense pain. Hence, treatment will have to include pain alleviation, regardless of the challenges in clinical pain diagnosis. The list of available drugs for treating pain in pigs is short, and pain is not a delimited indication. In this context, the following considerations will help generate a protocol for treating pain in pigs due to spontaneously occurring diseases and injuries.

Among the list of drugs for pigs, NSAIDs are commonly used in porcine health management and should be selected given the indication, location, nociceptive pathway and agent of concern [161]. Most commonly, meloxicam and ketoprofen are used as anti-inflammatory and analgesic drugs for farm animals [87, 163]. NSAIDs act at the periphery by targeting specific molecules involved in nociception in sensory neurons [2], and some have central analgesic effects [161]. NSAIDs have been found to be effective at alleviating inflammation but not neuropathic pain [164]. Depending on the injury of concern, experiences and recommendations for using NSAIDs for pigs in cases of mastitis [87, 165], lameness [166], incisional insult [41] or shoulder ulcer [42] have also been published.

Irrespective of the disease or injury, the half-life of a few hours for NSAIDs in pigs [167] requires the provision of more than one dose per day. Despite this limitation, the use of anti-inflammatory and analgesic agents is rated as the most effective method for reducing pain among animals [87, 168], and many studies have proven its efficacy for treating pain in pigs [105, 169, 170]. However, most NSAIDs are licenced to control pyrexia (Table 3), and few studies have examined the effective dose of NSAIDs for other indications [41, 161, 167]. Hence, further collaboration between researchers and practitioners concerning common adverse effects on healing [42] or even long-term effects are needed [161, 171].

**Table 3:** Licenced NSAIDs and related drugs in Germany

Agent	Dosage (mg/kg BW)	Application (times a day)	Application route	Maximum duration treatment (day)	References
Acetaminophen	30	1 [24 h]	P.O. (drinking water)	5	[172, 173]
Acetylsalicylic acid	30 50	2 1	P.O. (feed) P.O. (feed)	3 5–10	[174, 175]
Flunixin	2–2.2	1	IM	1–3	[176, 177]
Ketoprofen	1.5–3.0/ 3.0	1 [24 h]/ 1	P.O. (drinking water)/ IV, IM	1/ 1 (-3)	[178–180]
Meloxicam	0.4	1	IM	1 (-2)	[181–183]
Metamizole	15–50	Once/ 3 [8 h]	P.O./ IM, IV	1/ As needed	[184]
Sodium salicylate	35	1	P.O. (drinking water)	3–5	[185]
Tolfenamic acid	2.0	Once	IM	1	[186]

An example of licenced NSAIDs and related drugs in Germany for application in pigs (modified from VETIDATA, https://vetidata.de). For more information, refer to the search database of the European Medicines Agency (EMA, https://www.ema.europa.eu/en/medicines); max. maximum; P.O. Oral; IM Intramuscular; IV Intravenous; p8h every 8 h

In addition to NSAIDs, opioids can be successfully used to relieve (inflammatory) pain in model studies [38]. However, opioids are not licenced for general use in pigs [161]. In this respect, additional research is needed to broaden the range of potential drugs available for pigs, (see another discussion and overview with indications in [192]).

Importantly, the failure to treat acute (perioperative) pain may promote the emergence of peripheral and central sensitization and maladaptive pain conditions [187]. Nevertheless, given the understanding of the mechanisms of the different pain types, it seems rational that the administration of NSAIDs alone may be inadequate if maladaptive or chronic pain conditions can be assumed. In terms of a multimodal analgesic approach (i.e., the use of 2 or more analgesics or techniques to target different nociceptive pathways) [188], it would therefore be favourable to add adjunctive drugs to the therapy plan. Ketamine, an N-methyl-D-aspartate receptor (NMDA) antagonist, is administered at subanaesthetic doses and is known to modulate central sensitization and exert antihyperalgesic effects [187, 189]. However, ketamine is licenced for pigs for the purpose of injectable anaesthesia.

Closely related to enhancing the knowledge about the use of drugs for pain alleviation, knowledge about pain intensity is needed to evaluate the effect of analgesics. For practitioners, these data are relevant for attenuating the analgesic protocol. For example, a study found that the upper limit of mild pain scores and the diagnostic uncertainty zone overlap [190], indicating that pigs undergoing moderate pain should already receive analgesia [4]. However, even if no data for diagnostic zones are available in practice, the results of this study recommend initiating pain therapy when pain is identified by behavioural changes and/or on the basis of a diagnostic evaluation. In the latter case, it is not absolutely necessary to prove pain in the individual before starting the therapy. Pig veterinarians and farmers need to consider incorporating the judicious use of analgesics into standard operating procedures as a way of improving welfare [191]. In summary, monitoring the success of treatment is pivotal. Every practitioner should be aware that untreated or persistent pain can negatively affect health, welfare and quality of life. Nevertheless, in situations in which suffering and pain cannot be addressed, euthanasia should be regarded as the only viable option [187].

## Conclusion

Knowledge about the basic mechanisms, assessment and treatment of pain among pigs is needed to ensure that detrimental conditions among these animals are detected and alleviated in everyday practice. This article summarizes basic knowledge on this topic and invites readers to continue reading based on outlined references and topics. The paper provides guidance for practitioners based on the findings, details and intricacies of the latest research about pain in pigs. Limit of this article is that the search string included the term “not” and excluded the term “patients”.

More research about pain is necessary to increase knowledge about the diseases and injuries that veterinarians observe when examining pigs. One way to increase this knowledge is to link assumptions in research back to basic principles that drive every detrimental condition. Another way is to invite practitioners to provide more informed and detailed reports about treatment protocols for painful conditions in pigs. In this way, practice-research dialogue will help to obtain more evidence about pain induced by spontaneously occurring diseases and injuries in pigs.

### Short list for practitioners

- A pig that has been confirmed to be experiencing pain should receive adequate treatment
- Even a likely painful condition is enough reason to treat a pig for pain
- The fact that drugs are scarce, and pain identification is not easy does not justify leaving a pig suffering in a painful condition
- To identify pain among pigs, veterinarians and caretakers need deep knowledge about the basic principles of pain mechanisms; this article can be used as a starting point for narrowing knowledge gaps and identifying articles for further reading
- Scales and scores for identifying pain in pigs exist but need further validation in clinical settings; moreover, the current knowledge is sufficiently valid to prevent unnecessary pain in pigs

## Supplementary Information

Additional file 1. Metrics of the review. The table provides an overview of the search output and filter processes.

Additional file 2: Vocalization. The video shows a lame pig screaming while walking. Permission to reuse the materials for the purpose of illustrating the signs and arguments of the authors in this article is granted.

Additional file 3: Teeth grinding. The video shows a pig teeth grinding under environmental noise. Permission to reuse the materials for the purpose of illustrating the signs and arguments of the authors in this article is granted.

Additional file 4: Trembling. The video shows a pig with subtle trembling. Permission to reuse the materials for the purpose of illustrating the signs and arguments of the authors in this article is granted.

Additional file 5: Kneeling 1. The video shows a kneeling pig. Permission to reuse the materials for the purpose of illustrating the signs and arguments of the authors in this article is granted.

Additional file 6: Kneeling 2. The video shows a kneeling pig. Permission to reuse the materials for the purpose of illustrating the signs and arguments of the authors in this article is granted.

Additional file 7: Tipping. The video shows a tipping pig. Permission to reuse the materials for the purpose of illustrating the signs and arguments of the authors in this article is granted.

Additional file 8: Bending the spine. The picture shows a pig unburden a hind leg by bending the spine. Permission to reuse the materials for the purpose of illustrating the signs and arguments of the authors in this article is granted.

## Data Availability

No datasets were generated or analysed during the current study.

## References

[1] Hellebrekers LJ. Pathophysiology of pain in animals and its consequence for analgesic therapy. In: Hellebrekers LJ, editor., Animal Pain. A Practice-Oriented Approach to an Effective Pain Control in Animals. Utrecht, The Netherlands: Van der Wees; 2000. pp. 11–16.

[2] Vinuela-Fernandez I, Jones E, Welsh EM, Fleetwood-Walker SM. Pain mechanisms and their implication for the management of pain in farm and companion animals. Vet J. 2007;174(2):227–39. 10.1016/j.tvjl.2007.02.002. 17553712 10.1016/j.tvjl.2007.02.002

[3] Herskin MS, Di Giminiani P. Ch. 11 - Pain in pigs: Characterisation, mechanisms and indicators. In: Špinka M, editor. Advances in Pig Welfare: Woodhead Publishing; 2018. p. 325–55.

[4] Luna SPL, de Araújo AL, da Nóbrega Neto PI, Brondani JT, de Oliveira FA, Azerêdo LMDS, et al. Validation of the UNESP-Botucatu pig composite acute pain scale (UPAPS). PLoS ONE. 2020;15(6):e0233552-e. 10.1371/journal.pone.0233552. 32480399 10.1371/journal.pone.0233552 PMC7263847

[5] Marchant-Forde JN, Herskin MS. Ch. 16 - Pigs as laboratory animals. In: Špinka M, editor. Advances in Pig Welfare: Woodhead Publishing; 2018. p. 445–75.

[6] Prunier A, Tallet C, Sandercock DA. Evidence of pain in piglets subjected to invasisve management procedures. In: Edwards S, editor. Understanding the behaviour and improving the welfare of pigs. Cambridge: Burleigh dodds, Science publishing; 2021. p. 281–347.

[7] Sutherland MA. Welfare implications of invasive piglet husbandry procedures, methods of alleviation and alternatives: a review. N Z Vet J. 2015;63(1):52–7. 10.1080/00480169.2014.961990. 25204203 10.1080/00480169.2014.961990

[8] O’Connor A, Anthony R, Bergamasco L, Coetzee J, Gould S, Johnson AK, et al. Pain management in the neonatal piglet during routine management procedures. Part 2: grading the quality of evidence and the strength of recommendations. Anim Health Res Rev. 2014;15(1):39–62. 10.1017/S1466252314000073. 25605278 10.1017/S1466252314000073

[9] Dzikamunhenga RS, Anthony R, Coetzee J, Gould S, Johnson A, Karriker L, et al. Pain management in the neonatal piglet during routine management procedures. Part 1: a systematic review of randomized and non-randomized intervention studies. Anim Health Res Rev. 2014;15(1):14–38. 10.1017/S1466252314000061. 25605277 10.1017/S1466252314000061

[10] O’Connor A, Anthony R, Bergamasco L, Coetzee JF, Dzikamunhenga RS, Johnson AK, et al. Review: assessment of completeness of reporting in intervention studies using livestock: an example from pain mitigation interventions in neonatal piglets. Animal. 2016;10(4):660–70. 10.1017/S1751731115002323. 26556522 10.1017/S1751731115002323

[11] Contiero B, Cozzi G, Karpf L, Gottardo F. Pain in pig production: text mining analysis of the scientific literature. J Agric Environ Ethics. 2019;32(3):401–12. 10.1007/s10806-019-09781-4.

[12] Ison SH, Clutton RE, Di Giminiani P, Rutherford KM. A review of pain assessment in pigs. Front Vet Sci. 2016;3:108. 10.3389/fvets.2016.00108. 27965968 10.3389/fvets.2016.00108 PMC5124671

[13] Hewson CJ, Dohoo IR, Lemke KA, Barkema HW. Canadian veterinarians’ use of analgesics in cattle, pigs, and horses in 2004 and 2005. Can Vet J. 2007;48(2):155–64. 10.4141/cjas68-021. 17334029 10.4141/cjas68-021 PMC1780232

[14] Council directive No 98/58/EC (CD) of 20 July 1998. Protection of animals kept for farming purposes., (14.12.2019).

[15] Council regulation (EC) No 1099/2009, of 24 September 2009, on the protection of animals at the time of killing., (14.12.2019).

[16] Tricco AC, Lillie E, Zarin W, O’Brien KK, Colquhoun H, Levac D, et al. PRISMA extension for scoping reviews (PRISMA-ScR): checklist and explanation. Ann Intern Med. 2018;169(7):467–73. 10.7326/M18-0850. 30178033 10.7326/M18-0850

[17] Grosse Beilage E, Hennig-Pauka I, Kemper N, Kreienbrock L, Kunzmann P, Tölle K-H, et al. Abschlussbericht: Sofortmaßnahmen zur Vermeidung länger anhaltender erheblicher Schmerzen und Leiden bei schwer erkrankten/verletzten Schweinen durch rechtzeitige Tötung, 2021. Gießen: DVG Service. ISBN: 978–3–86345–609–2.

[18] Raja SN, Carr DB, Cohen M, Finnerup NB, Flor H, Gibson S, et al. The revised IASP definition of pain: Concepts, challenges, and compromises. PMC 2021; PMC7680716, NIHMSID: NIHMS1596925. 10.1097/j.pain.0000000000001939 PMC7680716 32694387

[19] Molony V, Kent JE. Assessment of acute pain in farm animals using behavioral and physiological measurements. J Anim Sci. 1997;75(1):266–72. 10.2527/1997.751266x. 9027575 10.2527/1997.751266x

[20] Meskey H, Bogduk N. Classification of chronic pain: Descriptions of chronic pain syndromes and definitions of pain terms. International Association for the Study of Pain (IASP), Seattle, 2nd edition. 1994.

[21] Gaynor JS, Muir WW. Handbook of veterinary pain management: Elsevier Health Sciences; 2014.

[22] Klinck MP, Troncy E. The physiology and pathophysiology of pain. BSAVA manual of canine and feline anaesthesia and analgesia: BSAVA Library; 2016. p. 97–112. 10.22233/9781910443231.8.

[23] Avon S, Wood R. Porcine skin as an in-vivo model for ageing of human bite marks. JFOS. 2005;23(2):30–9. 16353753

[24] Werland F, Hirth M, Rukwied R, Ringkamp M, Turnquist B, Jorum E, et al. Maximum axonal following frequency separates classes of cutaneous unmyelinated nociceptors in the pig. J Phsiol. 2021;599(5):1595–610. 10.1113/JP280269. 10.1113/JP280269 33369733

[25] Muir WW, Woolf CJ. Mechanisms of pain and their therapeutic implications. J Am Vet Med Assoc. 2001;219(10):1346–56. 11724168 10.2460/javma.2001.219.1346

[26] Shilo Y, Pascoe PJ. Anatomy, physiology, and pathophysiology of pain. In Egger CM, Love L, Doherty T (eds.), Pain Management in Veterinary Practice. 2013:9–27. 10.1002/9781118999196.ch2

[27] Smith ESJ, Lewin GR. Nociceptors: a phylogenetic view. J Comp Physiol A Neuroethol Sens Neural Behav Physiol. 2009;195(12):1089–106. 10.1007/s00359-009-0482-z. 19830434 10.1007/s00359-009-0482-z PMC2780683

[28] Costigan M, Scholz J, Woolf CJ. Neuropathic pain: a maladaptive response of the nervous system to damage. Annu Rev Neurosci. 2009;32:1–32. 10.1146/annurev.neuro.051508.135531. 19400724 10.1146/annurev.neuro.051508.135531 PMC2768555

[29] Vincent-Dospital T, Toussaint R, Måløy KJ. Heat emitting damage in skin: a thermal pathway for mechanical algesia. Front Neurosci. 2021;15: 780623. 10.3389/fnins.2021.780623. 34776861 10.3389/fnins.2021.780623 PMC8581405

[30] Sandercock DA, Coe JE, Di Giminiani P, Edwards SA. Determination of stable reference genes for RT-qPCR expression data in mechanistic pain studies on pig dorsal root ganglia and spinal cord. Res Vet Sci. 2017;114:493–501. 10.1016/j.rvsc.2017.09.025. 28987956 10.1016/j.rvsc.2017.09.025 PMC5667896

[31] Di Giminiani P, Edwards SA, Malcolm EM, Leach MC, Herskin MS, Sandercock DA. Characterization of short-and long-term mechanical sensitisation following surgical tail amputation in pigs. Sci Rep. 2017;7(1):4827. 10.1038/s41598-017-05404-y. 28684801 10.1038/s41598-017-05404-y PMC5500571

[32] Di Giminiani P, Sandercock DA, Malcolm EM, Leach MC, Herskin MS, Edwards SA. Application of a handheld pressure application measurement device for the characterisation of mechanical nociceptive thresholds in intact pig tails. Physiol Behav. 2016;165:119–26. 10.1016/j.physbeh.2016.07.006. 27422675 10.1016/j.physbeh.2016.07.006 PMC5038977

[33] Di Giminiani P, Stausholm JS, Viitasaari E, Petersen LJ, Herskin MS. The effect of social isolation, gender and familiarity with the experimental procedure on tests of porcine nociceptive thresholds. Vet Anaesth Analg. 2015;42(6):648–56. 10.1111/vaa.12254. 25752205 10.1111/vaa.12254

[34] Nalon E, Maes D, Piepers S, Taylor P, van Riet MM, Janssens GP, et al. Factors affecting mechanical nociceptive thresholds in healthy sows. Vet Anaesth Analg. 2016;43(3):343–55. 10.1111/vaa.12313. 26466760 10.1111/vaa.12313

[35] Di Giminiani P, Petersen LJ, Herskin MS. Nociceptive responses to thermal and mechanical stimulations in awake pigs. Eur J Pain. 2013;17(5):638–48. 10.1002/j.1532-2149.2012.00228.x. 23042703 10.1002/j.1532-2149.2012.00228.x

[36] Rettore Andreis F, Mørch CD, Jensen W, Meijs S. On determining the mechanical nociceptive threshold in pigs: a reliability study. Front Pain Res (Lausanne). 2023;4:1191786. 10.3389/fpain.2023.1191786. 37265942 10.3389/fpain.2023.1191786 PMC10229834

[37] Di Giminiani P, Petersen L, Herskin MS. Characterization of nociceptive behavioural responses in the awake pig following UV–B-induced inflammation. Eur J Pain. 2014;18(1):20–8. 10.1002/j.1532-2149.2013.00340.x. 23720380 10.1002/j.1532-2149.2013.00340.x

[38] Navarro-Alvarez N, Gonçalves BM, Andrews AR, Sachs DH, Huang CA. A CFA-induced model of inflammatory skin disease in miniature swine. Int J Inflam. 2018. 10.1155/2018/6916920. 30034774 10.1155/2018/6916920 PMC6035809

[39] Puyo CA, Earhart A, Staten N, Prince OA, Haug C, Kollef M, et al. Endotracheal intubation results in acute tracheal damage induced by mtDNA/TLR9/NF-κB activity. J Leukoc Biol. 2019;105(3):577–87. 10.1002/JLB.5A0718-254RR. 30548974 10.1002/JLB.5A0718-254RR PMC7379990

[40] Werland F, de Col R, Hirth M, Turnquist B, Schmelz M, Obreja O. Mechanical sensitization, increased axonal excitability, and spontaneous activity in C-nociceptors after ultraviolet B irradiation in pig skin. Pain. 2021;162(7):2002–13. 10.1097/j.pain.0000000000002197. 33449511 10.1097/j.pain.0000000000002197

[41] Levionnois O, Fosse T, Ranheim B. PK/PD modeling of flunixin meglumine in a kaolin-induced inflammation model in piglets. J Vet Pharmacol Ther. 2018;41(2):314–23. 10.1111/jvp.12468. 29143334 10.1111/jvp.12468

[42] Nystén M, Orro T, Peltoniemi O. Systemic inflammatory response to shoulder ulcers and lack of preventive effect of postpartum pain medication with ketoprofen in sows. Livest Sci. 2018;214:9–17. 10.1016/j.livsci.2018.04.019.

[43] Castel D, Sabbag I, Brenner O, Meilin S. Peripheral neuritis trauma in pigs: a neuropathic pain model. J Pain. 2016;17(1):36–49. 10.1016/j.jpain.2015.09.011. 26456763 10.1016/j.jpain.2015.09.011

[44] Castel D, Sabbag I, Nasaev E, Peng S, Meilin S. Open field and a behavior score in PNT model for neuropathic pain in pigs. J Pain Res. 2018. 10.2147/JPR.S172300. 30349354 10.2147/JPR.S172300 PMC6188018

[45] Sandercock DA, Barnett MW, Coe JE, Downing AC, Nirmal AJ, Di Giminiani P, et al. Transcriptomics analysis of porcine caudal dorsal root ganglia in tail amputated pigs shows long-term effects on many pain-associated genes. Front Vet Sci. 2019;18(6):314. 10.3389/fvets.2019.00314. 10.3389/fvets.2019.00314 PMC6760028 31620455

[46] Sandercock DA, Smith SH, Di Giminiani P, Edwards SA. Histopathological characterization of tail injury and traumatic neuroma development after tail docking in piglets. J Comp Pathol. 2016;155(1):40–9. 10.1016/j.jcpa.2016.05.003. 27302763 10.1016/j.jcpa.2016.05.003 PMC4940206

[47] Siddall PJ, Cousins MJ. Persistent pain as a disease entity: implications for clinical management. Anesth Analg. 2004;99(2):510–20. 10.1213/01.ANE.0000133383.17666.3A. 15271732 10.1213/01.ANE.0000133383.17666.3A

[48] Gasser HS, Erlanger J. The role of fiber size in the establishment of a nerve block by pressure or cocaine. Am J Physiol-Legacy Content. 1929;88(4):581–91. 10.1152/ajplegacy.1929.88.4.581.

[49] Self I, Grubb T. Physiology of pain. BSAVA Guide to Pain Management in Small Animal Practice: BSAVA Library; 2019. p. 3–13.

[50] Bosmans T, Doom M, Gasthuys F, Simoens P, Ham Lv, Polis I. Perioperative pain: physiology and pathophysiology. Vlaams Diergeneeskundig Tijdschrift. 2009;78(5):302–13.

[51] Bishop GH, Landau WM. Evidence for a double peripheral pathway for pain. Science. 1958;128(3326):712–3. 10.1126/science.128.3326.71. 13580241 10.1126/science.128.3326.712

[52] Ploner M, Gross J, Timmermann L, Schnitzler A. Cortical representation of first and second pain sensation in humans. Proc Natl Acad Sci USA. 2002;99(19):12444–8. 10.1073/pnas.182272899. 12209003 10.1073/pnas.182272899 PMC129464

[53] Cervero F. Sensory innervation of the viscera: peripheral basis of visceral pain. Physiol Rev. 1994;74(1):95–138. 10.1152/physrev.1994.74.1.95. 8295936 10.1152/physrev.1994.74.1.95

[54] Cervero F, Laird JM. Visceral pain. The Lancet. 1999;353(9170):2145–8. 10.1016/S0140-6736(99)01306-9. 10.1016/S0140-6736(99)01306-9 10382712

[55] Ison S, Rutherford K. Attitudes of farmers and veterinarians towards pain and the use of pain relief in pigs. Vet J. 2014;202(3):622–7. 10.1016/j.tvjl.2014.10.003. 25455386 10.1016/j.tvjl.2014.10.003

[56] Basbaum AI, Bautista DM, Scherrer G, Julius D. Cellular and molecular mechanisms of pain. Cell. 2009;139(2):267–84. 10.1016/j.cell.2009.09.028. 19837031 10.1016/j.cell.2009.09.028 PMC2852643

[57] Treede R-D, Rief W, Barke A, Aziz Q, Bennett MI, Benoliel R, et al. Chronic pain as a symptom or a disease: the IASP classification of chronic pain for the international classification of diseases (ICD-11). Pain. 2019;160(1):19–27. 10.1097/j.pain.0000000000001384. 30586067 10.1097/j.pain.0000000000001384

[58] Johnston CH, Whittaker AL, Franklin SH, Hutchinson MR. The neuroimmune interface and chronic pain through the lens of production animals. Front Neurosci. 2022;16: 887042. 10.3389/fnins.2022.887042. 35663552 10.3389/fnins.2022.887042 PMC9160236

[59] Adcock SJ. Early life painful procedures: long-term consequences and implications for farm animal welfare. Fron Anim Sci. 2021;2: 759522. 10.3389/fanim.2021.759522.

[60] Lindley S. Pain management III: chronic pain. BSAVA Manual of Canine and Feline Anaesthesia and Analgesia: BSAVA Library; 2016. p. 159–69.

[61] Cohen SP, Wang EJ, Doshi TL, Vase L, Cawcutt KA, Tontisirin N. Chronic pain and infection: mechanisms, causes, conditions, treatments, and controversies. BMJ Med. 2022;1(1): e000108. 10.1136/bmjmed-2021-000108. 36936554 10.1136/bmjmed-2021-000108 PMC10012866

[62] Kidd B, Urban L. Mechanisms of inflammatory pain. Br J Anaesth. 2001;87(1):3–11. 10.1093/bja/87.1.3. 11460811 10.1093/bja/87.1.3

[63] Baumgärtner W, Schmidt P. Entzündung. In: Baumgärtner W, Gruber AD, (editors). Allgemeine Pathologie für die Tiermedizin. 3., aktualisierte Auflage ed: Georg Thieme Verlag; 2020. 10.1055/b-0040-175119.

[64] Lemke KA. Understanding the pathophysiology of perioperative pain. The Can Vet J. 2004;45(5):405–13. 15206589 PMC548624

[65] Chiu IM, Von Hehn CA, Woolf CJ. Neurogenic inflammation and the peripheral nervous system in host defense and immunopathology. Nat Neurosci. 2012;15(8):1063–7. 10.1038/nn.3144. 22837035 10.1038/nn.3144 PMC3520068

[66] Pinho-Ribeiro FA, Verri WA, Chiu IM. Nociceptor sensory neuron–immune interactions in pain and inflammation. Trends in immunol. 2017;38(1):5–19. 10.1016/j.it.2016.10.001. 27793571 10.1016/j.it.2016.10.001 PMC5205568

[67] Enomoto M, Mantyh PW, Murrell J, Innes JF, Lascelles BDX. Anti-nerve growth factor monoclonal antibodies for the control of pain in dogs and cats. Vet Rec. 2019;184(1):23. 10.1136/vr.104590. 30368458 10.1136/vr.104590 PMC6326241

[68] Lascelles BDX, Knazovicky D, Case B, Freire M, Innes JF, Drew AC, et al. A canine-specific anti-nerve growth factor antibody alleviates pain and improves mobility and function in dogs with degenerative joint disease-associated pain. BMC Vet Res. 2015. 10.1186/s12917-015-0413-x. 25926287 10.1186/s12917-015-0413-x PMC4419463

[69] Gruen M, Thomson A, Griffith E, Paradise H, Gearing D, Lascelles B. A feline-specific anti-nerve growth factor antibody improves mobility in cats with degenerative joint disease–associated pain: A pilot proof of concept study. J Vet Intern Med. 2016;30(4):1138–48. 10.1111/jvim.13972. 27334504 10.1111/jvim.13972 PMC5153962

[70] Jensen MP, Chodroff MJ, Dworkin RH. The impact of neuropathic pain on health-related quality of life: review and implications. Neurology. 2007;68(15):1178–82. 10.1212/01.wnl.0000259085.61898.9e. 17420400 10.1212/01.wnl.0000259085.61898.9e

[71] Latremoliere A, Woolf CJ. Central sensitization: a generator of pain hypersensitivity by central neural plasticity. J Pain. 2009;10(9):895–926. 10.1016/j.jpain.2009.06.012. 19712899 10.1016/j.jpain.2009.06.012 PMC2750819

[72] White K, Hunt J. Chronic and osteoarthritic pain. BSAVA Guide to Pain Management in Small Animal Practice: BSAVA Library; 2019. p. 24–41.

[73] Hucho T, Levine JD. Signaling pathways in sensitization: toward a nociceptor cell biology. Neuron. 2007;55(3):365–76. 10.1016/j.neuron.2007.07.008. 17678851 10.1016/j.neuron.2007.07.008

[74] Woolf CJ. Central sensitization: implications for the diagnosis and treatment of pain. Pain. 2011;152(3):2–15. 10.1016/j.pain.2010.09.030. 20961685 10.1016/j.pain.2010.09.030 PMC3268359

[75] Neumann S, Doubell TP, Leslie T, Woolf CJ. Inflammatory pain hypersensitivity mediated by phenotypic switch in myelinated primary sensory neurons. Nature. 1996;384(6607):360–4. 10.1038/384360a0. 8934522 10.1038/384360a0

[76] Herrero JF, Laird JM, Lopez-Garcia JA. Wind-up of spinal cord neurones and pain sensation: much ado about something? Prog Neurobiol. 2000;61(2):169–203. 10.1016/s0301-0082(99)00051-9. 10704997 10.1016/s0301-0082(99)00051-9

[77] Heinonen M, Orro T, Kokkonen T, Munsterhjelm C, Peltoniemi O, Valros A. Tail biting induces a strong acute phase response and tail-end inflammation in finishing pigs. Vet J. 2010;184(3):303–7. 10.1016/j.tvjl.2009.02.021. 19398209 10.1016/j.tvjl.2009.02.021

[78] Raffaeli W, Arnaudo E. Pain as a disease: an overview. J Pain Res. 2017. 10.2147/JPR.S138864. 28860855 10.2147/JPR.S138864 PMC5573040

[79] Sandercock D, Smith S, Di Giminiani P, Edwards S. Corrigendum to histopathological characterization of tail injury and traumatic neuroma development after tail docking in piglets. J Comp Pathol. 2016;155(2–3):276. 10.1016/j.jcpa.2016.08.002. 27302763 10.1016/j.jcpa.2016.05.003 PMC4940206

[80] Janczak AM, Ranheim B, Fosse TK, Hild S, Nordgreen J, Moe RO, et al. Factors affecting mechanical (nociceptive) thresholds in piglets. Vet Anaesth Analg. 2012;39(6):628–35. 10.1111/j.1467-2995.2012.00737.x. 22709378 10.1111/j.1467-2995.2012.00737.x PMC3586661

[81] Diana A, Carpentier L, Piette D, Boyle LA, Berckmans D, Norton T. An ethogram of biter and bitten pigs during an ear biting event: first step in the development of a Precision Livestock Farming tool. Appl Anim Behav Sci. 2019;215:26–36. 10.1016/j.applanim.2019.03.011.

[82] Leslie E, Hernández-Jover M, Newman R, Holyoake P. Assessment of acute pain experienced by piglets from ear tagging, ear notching and intraperitoneal injectable transponders. Appl Anim Behav Sci. 2010;127(3–4):86–95. 10.1016/j.applanim.2010.09.006.

[83] Rutherford KM, Thompson CS, Thomson JR, Lawrence AB, Nielsen EO, Busch ME, et al. A study of associations between gastric ulcers and the behaviour of finisher pigs. Livest Sci. 2018;212:45–51. 10.1016/j.livsci.2018.03.013.

[84] Osther PJ, Pedersen KV, Lildal SK, Pless MS, Andreassen KH, Osther SS, et al. Pathophysiological aspects of ureterorenoscopic management of upper urinary tract calculi. Curr Opin Urol. 2016;26(1):63–9. 10.1097/MOU.0000000000000235. 26555686 10.1097/MOU.0000000000000235

[85] Mozzachio K, Tynes VV. Recognition and treatment of pain in pet pigs. In Egger, CM, Love L, Doherty, T (editors): Pain Management in Veterinary Practice. 2013:383–9. 10.1002/9781118999196.ch34.

[86] Flecknell P. Analgesia from a veterinary perspective. Br J Anaesth. 2008;101(1):121–4. 10.1093/bja/aen087. 18424804 10.1093/bja/aen087

[87] Wilson R, Holyoake P, Cronin G, Doyle R. Managing animal wellbeing: a preliminary survey of pig farmers. Aust Vet J. 2014;92(6):206–12. 10.1111/avj.12169. 24731237 10.1111/avj.12169

[88] Tomacheuski RM, Monteiro BP, Evangelista MC, Luna SPL, Steagall PV. Measurement properties of pain scoring instruments in farm animals: A systematic review using the COSMIN checklist. PLoS ONE. 2023;18(1): e0280830. 10.1371/journal.pone.0280830. 36662813 10.1371/journal.pone.0280830 PMC9858734

[89] Grosse Beilage E, Wendt M. Diagnostik und Gesundheitsmanagement im Schweinebestand. Verlag Eugen Ulmer Stuttgart/UTB, ISBN 978–3–8252–8502–9. 2013.

[90] Broom DM. Animal welfare: concepts and measurement. J Anim Sci. 1991;69(10):4167–75. 10.2527/1991.69104167x. 1778832 10.2527/1991.69104167x

[91] Zonderland JJ, van Riel JW, Bracke MBM, Kemp B, den Hartog LA, Spoolder HAM. Tail posture predicts tail damage among weaned piglets. Appl Anim Behav Sci. 2009;121(3):165–70. 10.1016/j.applanim.2009.09.002.

[92] Reid J, Nolan A, Hughes L, Lascelles BDX, Pawson P, Scott EM. Development of the short-form glasgow composite measure pain scale (CMPS-SF) and derivation of an analgesic intervention score. Anim Welf. 2007;16:97–104. 10.1017/S096272860003178X.

[93] Bussières G, Jacques C, Lainay O, Beauchamp G, Leblond A, Cadoré JL, et al. Development of a composite orthopaedic pain scale in horses. Res Vet Sci. 2008;85(2):294–306. 10.1016/j.rvsc.2007.10.011. 18061637 10.1016/j.rvsc.2007.10.011

[94] Sutton GA, Atamna R, Steinman A, Mair TS. Comparison of three acute colic pain scales: reliability, validity and usability. Vet J. 2019;246:71–7. 10.1016/j.tvjl.2019.01.004. 30902193 10.1016/j.tvjl.2019.01.004

[95] Boesch JM, Roinestad KE, Lopez DJ, Newman AK, Campoy L, Gleed RD, et al. The canine postamputation pain (CAMPPAIN) initiative: a retrospective study and development of a diagnostic scale. Vet Anaesth Analg. 2021;48(6):861–70. 10.1016/j.vaa.2021.07.003. 34483040 10.1016/j.vaa.2021.07.003

[96] Larsen T, Kaiser M, Herskin MS. Does the presence of shoulder ulcers affect the behaviour of sows? Res Vet Sci. 2015;98:19–24. 10.1016/j.rvsc.2014.11.001. 25468797 10.1016/j.rvsc.2014.11.001

[97] da Silva Cordeiro AF, de Alencar NI, Oliveira SR, Violaro F, de Almeida AC, Neves DP. Understanding vocalization might help to assess stressful conditions in piglets. Animals (Basel). 2013;3(3):923–34. 10.3390/ani3030923. 26479541 10.3390/ani3030923 PMC4494434

[98] Marx G, Horn T, Thielebein J, Knubel B, von Borell E. Analysis of pain-related vocalization in young pigs. J Sound Vib. 2003;266(3):687–98. 10.1016/S0022-460X(03)00594-7.

[99] Di Giminiani P, Nasirahmadi A, Malcolm EM, Leach MC, Edwards SA. Docking piglet tails: How much does it hurt and for how long? Physiol & behav. 2017;182:69–76. 10.1016/j.physbeh.2017.09.028. 28974458 10.1016/j.physbeh.2017.09.028

[100] Oldham JG. Clinical measurement of pain, distress and discomfort in pigs. In: Gibson TE, editor. The Detection and Relief of Pain in Animals. London: BVA Animal Welfare Foundation; 1985. p. 88–90.

[101] Kluivers-Poodt M, Zonderland JJ, Verbraak J, Lambooij E, Hellebrekers LJ. Pain behaviour after castration of piglets; effect of pain relief with lidocaine and/or meloxicam. Animal. 2013;7(7):1158–62. 10.1017/S1751731113000086. 23388116 10.1017/S1751731113000086

[102] Camerlink I, Ursinus WW. Tail postures and tail motion in pigs: A review. Appl Anim Behav Sci. 2020;230: 105079. 10.1016/j.applanim.2020.105079.

[103] Wedin M, Baxter EM, Jack M, Futro A, D’Eath RB. Early indicators of tail biting outbreaks in pigs. Appl Anim Behav Sci. 2018;208:7–13. 10.1016/j.applanim.2018.08.008.

[104] Conte S, Bergeron R, Gonyou H, Brown J, Rioja-Lang FC, Connor L, et al. Measure and characterization of lameness in gestating sows using force plate, kinematic, and accelerometer methods. J Anim Sci. 2014;92(12):5693–703. 10.2527/jas.2014-7865. 25403203 10.2527/jas.2014-7865

[105] Meijer E, van Nes A, Back W, van der Staay FJ. Clinical effects of buprenorphine on open field behaviour and gait symmetry in healthy and lame weaned piglets. Vet J. 2015;206(3):298–303. 10.1016/j.tvjl.2015.10.016. 26521014 10.1016/j.tvjl.2015.10.016

[106] Grégoire J, Bergeron R, D’Allaire S, Meunier-Salaün MC, Devillers N. Assessment of lameness in sows using gait, footprints, postural behaviour and foot lesion analysis. Animal. 2013;7(7):1163–73. 10.1017/S1751731113000098. 23391233 10.1017/S1751731113000098

[107] Weary DM, Niel L, Flower FC, Fraser D. Identifying and preventing pain in animals. Appl Anim Behav Sci. 2006;100(1):64–76. 10.1016/j.applanim.2006.04.013.

[108] Dabareiner R, Carter K, Dyson S. The tarsus. In: Ross MW, Dyson SJ, editors. Diagnosis and Management of Lameness in the Horse: Elsevier Health Sciences; 2010. p. 440–92.

[109] Di Giminiani P, Brierley VL, Scollo A, Gottardo F, Malcolm EM, Edwards SA, et al. The assessment of facial expressions in piglets undergoing tail docking and castration: toward the development of the piglet grimace scale. Front Vet Sci. 2016;3:100. 10.3389/fvets.2016.00100. 27896270 10.3389/fvets.2016.00100 PMC5107875

[110] Viscardi AV, Hunniford M, Lawlis P, Leach M, Turner PV. Development of a piglet grimace scale to evaluate piglet pain using facial expressions following castration and tail docking: a pilot study. Front Vet Sci. 2017. 10.3389/fvets.2017.00051. 28459052 10.3389/fvets.2017.00051 PMC5394162

[111] Vullo C, Barbieri S, Catone G, Graïc JM, Magaletti M, Di Rosa A, et al. Is the piglet grimace scale (PGS) a useful welfare indicator to assess pain after cryptorchidectomy in growing pigs? Animals (Basel). 2020;10(3):412. 10.3390/ani10030412. 32131424 10.3390/ani10030412 PMC7143901

[112] Navarro E, Mainau E, Manteca X. Development of a facial expression scale using farrowing as a model of pain in sows. Animals (Basel). 2020;10(11):2113. 10.3390/ani10112113. 33202526 10.3390/ani10112113 PMC7696890

[113] Lezama-García K, Orihuela A, Olmos-Hernández A, Reyes-Long S, Mota-Rojas D. Facial expressions and emotions in domestic animals. CABI Rev. 2019;2019:1–12. 10.1079/PAVSNNR201914028.

[114] Sneddon LU, Elwood RW, Adamo SA, Leach MC. Defining and assessing animal pain. Anim Behav. 2014;97:201–12. 10.1016/j.anbehav.2014.09.007.

[115] Lou ME, Porter ST, Massey JS, Ventura B, Deen J, Li Y. The application of 3D landmark-based geometric morphometrics towards refinement of the piglet grimace scale. Animals (Basel). 2022;12(15):1944. 10.3390/ani12151944. 35953933 10.3390/ani12151944 PMC9367447

[116] Lencioni GC, de Sousa RV, de Souza Sardinha EJ, Corrêa RR, Zanella AJ. Pain assessment in horses using automatic facial expression recognition through deep learning-based modeling. PLoS ONE. 2021;16(10): e0258672. 10.1371/journal.pone.0258672. 34665834 10.1371/journal.pone.0258672 PMC8525760

[117] Feighelstein M, Henze L, Meller S, Shimshoni I, Hermoni B, Berko M, et al. Explainable automated pain recognition in cats. Sci Rep. 2023;13(1):8973. 10.1038/s41598-023-35846-6. 37268666 10.1038/s41598-023-35846-6 PMC10238514

[118] Feighelstein M, Ehrlich Y, Naftaly L, Alpin M, Nadir S, Shimshoni I, et al. Deep learning for video-based automated pain recognition in rabbits. Sci Rep. 2023;13(1):14679. 10.1038/s41598-023-41774-2. 37674052 10.1038/s41598-023-41774-2 PMC10482887

[119] Neethirajan S. Happy cow or thinking pig? Wur wolf—facial coding platform for measuring emotions in farm animals. AI. 2021;2(3):342–54. 10.3390/ai2030021.

[120] Henke J, Tacke S, Erhardt W. Analgesie. W Erhardt, J Henke, J Haberstroh, C Baumgartner, S Tacke (Hrsg): Anästhesie und Analgesie beim Klein- und Heimtier. 2. Auflage ed2012. p. 383–434.

[121] Le Bars D, Gozariu M, Cadden SW. Animal models of nociception. Pharmacol Rev. 2001;53(4):597–652. 11734620

[122] Antognini JF, Barter L, Carstens E. Movement as an index of anesthetic depth in humans and experimental animals. Comp Med. 2005;55(5):413–8. 16270896

[123] Prunier A, Mounier A, Hay M. Effects of castration, tooth resection, or tail docking on plasma metabolites and stress hormones in young pigs. J Anim Sci. 2005;83(1):216–22. 10.2527/2005.831216x. 15583062 10.2527/2005.831216x

[124] Prunier A, Mounier L, Le Neindre P, Leterrier C, Mormède P, Paulmier V, et al. Identifying and monitoring pain in farm animals: a review. Animal. 2013;7(6):998–1010. 10.1017/S1751731112002406. 23254122 10.1017/S1751731112002406

[125] Gottardo F, Scollo A, Contiero B, Ravagnani A, Tavella G, Bernardini D, et al. Pain alleviation during castration of piglets: a comparative study of different farm options. J Anim Sci. 2016;94(12):5077–88. 10.2527/jas.2016-0843. 28046151 10.2527/jas.2016-0843

[126] Mück S. Belastung von Schweinen bei Therapie mit einem mehrmalig im Vergleich zu einem einmalig zu injizierenden Präparat [Diss.]. München: Ludwig-Maximilians-Universität München; 2017. 10.5282/edoc.21237.

[127] Contreras-Aguilar MD, Escribano D, Martínez-Miró S, López-Arjona M, Rubio CP, Martínez-Subiela S, et al. Application of a score for evaluation of pain, distress and discomfort in pigs with lameness and prolapses: correlation with saliva biomarkers and severity of the disease. Res Vet Sci. 2019;126:155–63. 10.1016/j.rvsc.2019.08.004. 31494378 10.1016/j.rvsc.2019.08.004

[128] Mohling CM, Pairis-Garcia MD, Johnson AK, Stalder KJ, Karriker LA, Coetzee JF, et al. Blood cortisol as an objective tool to measure painful and non-painful hoof lameness states in multiparous sows. Iowa State Univ Animal Industry Report. 2013. 10.31274/ans_air-180814-863.

[129] Carroll G, Boyle L, Hanlon A, Palmer M, Collins L, Griffin K, et al. Identifying physiological measures of lifetime welfare status in pigs: exploring the usefulness of haptoglobin, C-reactive protein and hair cortisol sampled at the time of slaughter. Ir Vet J. 2018;71:1–10. 10.1186/s13620-018-0118-0. 29507716 10.1186/s13620-018-0118-0 PMC5833096

[130] Prims S, Hole CV, Van Cruchten S, Van Ginneken C, Van Ostade X, Casteleyn C. Hair or salivary cortisol analysis to identify chronic stress in piglets? Vet J. 2019;252: 105357. 10.1016/j.tvjl.2019.105357. 31554592 10.1016/j.tvjl.2019.105357

[131] Sann H. Nozizeption und Schmerz. In: Breves, G, M Diener, G Gäbel et al (editors): Physiologie der Haustiere. 5. Auflage ed. Stuttgart: Enke Verlag; 2015. p. 78–84. 10.1055/b-0042-188492.

[132] Leliavski A, Dumbell R, Ott V, Oster H. Adrenal clocks and the role of adrenal hormones in the regulation of circadian physiology. J Biol Rhythms. 2015;30(1):20–34. 10.1177/0748730414553971. 25367898 10.1177/0748730414553971

[133] Lonardi C, Scollo A, Normando S, Brscic M, Gottardo F. Can novel methods be useful for pain assessment of castrated piglets? Animal. 2015;9(5):871–7. 10.1017/S1751731114003176. 25557652 10.1017/S1751731114003176

[134] Bova JF, Da Cunha AF, Stout RW, Bhumiratana S, Alfi DM, Eisig SB, et al. Bupivacaine mandibular nerve block affects intraoperative blood pressure and heart rate in a Yucatan miniature swine mandibular condylectomy model: a pilot study. J Invest Surg. 2015;28(1):32–9. 10.3109/08941939.2014.971207. 25394295 10.3109/08941939.2014.971207 PMC4479207

[135] Leitão CJ, Lima-Rodríguez JR, Ferreira F, Avelino C, Sánchez-Margallo FM, Antunes L. Parasympathetic tone activity evaluation to discriminate ketorolac and ketorolac/tramadol analgesia level in swine. Anesth, Analg. 2019;129(3):882–9. 10.1213/ANE.0000000000003573. 31425233 10.1213/ANE.0000000000003573

[136] Saller AM, Werner J, Reiser J, Senf S, Deffner P, Abendschön N, et al. Local anesthesia in piglets undergoing castration—A comparative study to investigate the analgesic effects of four local anesthetics on the basis of acute physiological responses and limb movements. PLoS ONE. 2020;15(7): e0236742. 10.1371/journal.pone.0236742. 32730302 10.1371/journal.pone.0236742 PMC7392247

[137] Söbbeler FJ, Wendt S, Briese A, Tünsmeier J, Waldmann K-H, Kästner SBR, et al. Comparative study of pain-related responses of male piglets up to seven days of age to the application of different local anaesthetics and subsequent castration. Animals (Basel). 2022;12(20):2833. 10.3390/ani12202833. 36290223 10.3390/ani12202833 PMC9597853

[138] Gottardo F, Scollo A, Contiero B, Ravagnani A, Tavella G, Bernardini D, et al. Pain alleviation during castration of piglets: a comparative study of different farm options. J Anim Sc. 2016;94(12):5077–88. 10.2527/jas.2016-0843. 28046151 10.2527/jas.2016-0843

[139] Bonastre C, Mitjana O, Tejedor M, Calavia M, Yuste A, Úbeda J, et al. Acute physiological responses to castration-related pain in piglets: the effect of two local anesthetics with or without meloxicam. Animal. 2016;10(9):1474–81. 10.1017/S1751731116000586. 27080170 10.1017/S1751731116000586

[140] Temple D, Escribano D, Jiménez M, Mainau E, Cerón JJ, Manteca X. Effect of the needle-free “intra dermal application of liquids” vaccination on the welfare of pregnant sows. Porcine Health Manag. 2017;3:1–7. 10.1186/s40813-017-0056-3. 28405465 10.1186/s40813-017-0056-3 PMC5382458

[141] Hofmann K, Rauh A, Harlizius J, Weiß C, Scholz T, Schulze-Horsel T, et al. Schmerz-und Stressbestimmung bei der Injektion und Kastration von Saugferkeln unter Lokalanästhesie mit Procain und Lidocain. Teil 1: Kortisol, Chromogranin A, Wundheilung, Gewichtsentwicklung, Saugferkelverluste. Tierärztl Prax Ausg G Grosstiere Nutztiere. 2019;47(02):87–96. 10.1055/a-0861-9640. 30999349 10.1055/a-0861-9640

[142] Rauh A, Hofmann K, Harlizius J, Weiß C, Numberger J, Scholz T, et al. Schmerz-und Stressbestimmung bei der Injektion und Kastration von Saugferkeln unter Lokalanästhesie mit Procain und Lidocain. Teil 2: Abwehrverhalten, Katecholamine, koordinierte Bewegungsabläufe. Tierarztl Prax Ausg G Grosstiere Nutztiere. 2019;47(03):160–70. 10.1055/a-0866-6694. 31212342 10.1055/a-0866-6694

[143] Sutherland M, Davis B, Brooks T, Coetzee J. The physiological and behavioral response of pigs castrated with and without anesthesia or analgesia. J Anim Sci. 2012;90(7):2211–21. 10.2527/jas.2011-4260. 22266989 10.2527/jas.2011-4260

[144] Bates JL, Karriker LA, Stock ML, Pertzborn KM, Baldwin LG, Wulf LW, et al. Impact of transmammary-delivered meloxicam on biomarkers of pain and distress in piglets after castration and tail docking. PLoS ONE. 2014;9(12): e113678. 10.1371/journal.pone.0113678. 25437866 10.1371/journal.pone.0113678 PMC4249978

[145] Hansson M, Lundeheim N, Nyman G, Johansson G. Effect of local anaesthesia and/or analgesia on pain responses induced by piglet castration. Acta Vet Scand. 2011;53(1):34. 10.1186/1751-0147-53-34. 21627797 10.1186/1751-0147-53-34 PMC3123560

[146] Temple D, Jiménez M, Escribano D, Martín-Valls G, Díaz I, Manteca X. Welfare benefits of intradermal vaccination of piglets. Animals. 2020;10(10):1898. 10.3390/ani10101898. 33081216 10.3390/ani10101898 PMC7602853

[147] Kluivers-Poodt M, Houx B, Robben S, Koop G, Lambooij E, Hellebrekers L. Effects of a local anaesthetic and NSAID in castration of piglets, on the acute pain responses, growth and mortality. Animal. 2012;6(9):1469–75. 10.1017/S1751731112000547. 23031520 10.1017/S1751731112000547

[148] Tecles F, Contreras-Aguilar MD, Martínez-Miró S, Tvarijonaviciute A, Martínez-Subiela S, Escribano D, et al. Total esterase measurement in saliva of pigs: Validation of an automated assay, characterization and changes in stress and disease conditions. Res Vet Sci. 2017;114:170–6. 10.1016/j.rvsc.2017.04.007. 28441610 10.1016/j.rvsc.2017.04.007

[149] Maršálek P, Svoboda M, Smutná M, Blahová J, Večerek V. Neopterin and biopterin as biomarkers of immune system activation associated with castration in piglets. J Anim Sci. 2011;89(6):1758–62. 10.2527/jas.2010-3157. 21297064 10.2527/jas.2010-3157

[150] Maršálek P, Svoboda M, Bernardy J, Večerek V. Concentrations of neopterin, biopterin, and cortisol associated with surgical castration of piglets with lidocaine Original Paper. Czech J Anim Sci. 2015;60(11):473–8. 10.17221/8555-CJAS.

[151] Lykkegaard K, Lykkesfeldt J, Lauritzen B, Svendsen O. Morphine reduces spinal c-fos expression dose-dependently during experimental laparotomy in pigs: a combined pharmacokinetic and surgical study. Res Vet Sci. 2008;84(3):457–64. 10.1016/j.rvsc.2007.06.001. 17675197 10.1016/j.rvsc.2007.06.001

[152] Reiser J, Kreuzer M, Werner J, Saller AM, Fischer J, Senf S, et al. Nociception-induced changes in electroencephalographic activity and FOS protein expression in piglets undergoing castration under isoflurane anaesthesia. Animals (Basel). 2022;12(18):2309. 10.3390/ani12182309. 36139169 10.3390/ani12182309 PMC9494976

[153] Haga HA, Ranheim B. Castration of piglets: the analgesic effects of intratesticular and intrafunicular lidocaine injection. Vet Anaesth Analg. 2005;32(1):1–9. 10.1111/j.1467-2995.2004.00225.x. 15663733 10.1111/j.1467-2995.2004.00225.x

[154] Kells NJ, Beausoleil NJ, Chambers JP, Sutherland MA, Morrison RS, Johnson CB. Electroencephalographic responses of anaesthetized pigs (Sus scrofa) to tail docking using clippers or cautery iron performed at 2 or 20 days of age. Vet Anaesth Analg. 2017;44(5):1156–65. 10.1016/j.vaa.2017.02.003. 28412163 10.1016/j.vaa.2017.02.003

[155] Kells NJ, Beausoleil NJ, Sutherland M. Post-natal development of EEG responses to noxious stimulation in pigs Sus scrofa aged 1–15 days. Anim Welf. 2019;28:317–29. 10.7120/09627286.28.3.317.

[156] Mota-Rojas D, Olmos-Hernández A, Verduzco-Mendoza A, Lecona-Butrón H, Martínez-Burnes J, Mora-Medina P, et al. Infrared thermal imaging associated with pain in laboratory animals. Exp Anim. 2021;70(1):1–12. 10.1538/expanim.20-0052. 32848100 10.1538/expanim.20-0052 PMC7887630

[157] Staveley LM, Zemitis JE, Plush KJ, D’Souza DN. Infrared thermography for early identification and treatment of shoulder sores to improve sow and piglet welfare. Animals (Basel). 2022;12(22):3136. 10.3390/ani12223136. 36428364 10.3390/ani12223136 PMC9686874

[158] Haussler KK. Pressure algometry for the detection of mechanical nociceptive thresholds in horses. Animals (Basel). 2020;10(12):2195. 10.3390/ani10122195. 33255216 10.3390/ani10122195 PMC7760268

[159] Haussler KK, Hill AE, Frisbie DD, McIlwraith CW. Determination and use of mechanical nociceptive thresholds of the thoracic limb to assess pain associated with induced osteoarthritis of the middle carpal joint in horses. AM J Vet Res. 2007;68(11):1167–76. 10.2460/ajvr.68.11.1167. 17975970 10.2460/ajvr.68.11.1167

[160] Kornum BR, Knudsen GM. Cognitive testing of pigs (Sus scrofa) in translational biobehavioral research. Neurosci Biobehav Rev. 2011;35(3):437–51. 10.1016/j.neubiorev.2010.05.004. 20553757 10.1016/j.neubiorev.2010.05.004

[161] Schoos A, Devreese M, Maes DG. Use of non-steroidal anti-inflammatory drugs in porcine health management. Vet Rec. 2019;185(6):172. 10.1136/vr.105170. 31040220 10.1136/vr.105170

[162] Viitasaari E, Raekallio M, Valros A, Peltoniemi O, Hänninen L, Heinonen M. The effect of ketoprofen on feeding behavior of tail-bitten pigs. Porc Health Manag. 2015;1:1–7. 10.1186/s40813-015-0005-y.

[163] Viscardi A, Turner P. Use of meloxicam, buprenorphine, and Maxilene® to assess a multimodal approach for piglet pain management, part 1: surgical castration. Anim Welf. 2019;28:487–98. 10.7120/09627286.28.4.487.

[164] Reyes L, Tinworth KD, Li KM, Yau DF, Waters KA. Observer-blinded comparison of two nonopioid analgesics for postoperative pain in piglets. Pharmacol Biochem Behav. 2002;73(3):521–8. 10.1016/s0091-3057(02)00820-1. 12151025 10.1016/s0091-3057(02)00820-1

[165] Hirsch AC, Philipp H, Kleemann R. Investigation on the efficacy of meloxicam in sows with mastitis–metritis–agalactia syndrome. J Vet Pharmacol Ther. 2003;26(5):355–60. 10.1046/j.1365-2885.2003.00524.x. 14633188 10.1046/j.1365-2885.2003.00524.x

[166] Pairis-Garcia M, Johnson A, Abell C, Coetzee J, Karriker L, Millman S, et al. Measuring the efficacy of flunixin meglumine and meloxicam for lame sows using a GAITFour pressure mat and an embedded microcomputer-based force plate system. J Anim Sci. 2015;93(5):2100–10. 10.2527/jas.2014-8796. 26020306 10.2527/jas.2014-8796

[167] Karriker LA, Coetzee JF, Friendship RM, Apley MD. Drug pharmocology, therapy and prophylaxis. In: Zimmermann JJ, Karriker, LA, Ramirez, A., Schwartz, K.J., Stevenson, GW, Zhang, J, (editors). In: Diseases of Swine, ed.11^th^, Wiley Blackwell; 2019. 10.1002/9781119350927.ch10.

[168] Keita A, Pagot E, Prunier A, Guidarini C. Pre-emptive meloxicam for postoperative analgesia in piglets undergoing surgical castration. Vet Anaesth Analg. 2010;37(4):367–74. 10.1111/j.1467-2995.2010.00546.x. 20636569 10.1111/j.1467-2995.2010.00546.x

[169] Conte S, Bergeron R, Gonyou H, Brown J, Rioja-Lang FC, Connor ML, et al. Use of an analgesic to identify pain-related indicators of lameness in sows. Livest Sci. 2015;180:203–8. 10.1016/j.livsci.2015.08.009.

[170] Whalin L, Pairis-Garcia M, Proudfoot K, Stalder K, Johnson A. Validating behavioral sampling techniques for lame sows administered flunixin meglumine and meloxicam. Livest Sci. 2016;191:103–7. 10.1016/j.livsci.2016.07.017.

[171] Gorissen BMC, Uilenreef JJ, Bergmann W, Meijer E, van Rietbergen B, van der Staay FJ, et al. Effects of long-term use of the preferential COX-2 inhibitor meloxicam on growing pigs. Veterinary Record. 2017;181(21):564. 10.1136/vr.104175. 29066475 10.1136/vr.104175

[172] Ceva Tiergesundheit GmbH. Summary of Product Characteristics Pracetam® 200 mg/ml Pulver zum Eingeben über das Trinkwasser für Schweine. 2015.

[173] Ceva Tiergesundheit GmbH. Summary of Product Characteristics Pracetam® 400 mg/ml Lösung zum Eingeben über das Trinkwasser für Schweine. 2021.

[174] aniMedica GmbH. Summary of Product Characteristics Suispirin, 1000mg/g, oral powder for pigs. 2016.

[175] Veyx Pharma GmbH. Summary of Product Characteristics Pyrinagil 100 % Pulver zum Eingeben für Schweine. 2014.

[176] Intervet Deutschland GmbH. Summary of Product Characteristics Finadyne RPS 83 mg/ml Injektionslösung für Rinder, Pferde und Schweine. 2019.

[177] Ceva Tiergesundheit GmbH. Summary of Product Characteristics Wellicox 50 mg /ml Injektionslösung für Rinder, Schweine und Pferde. 2020.

[178] Ecuphar Veterinaria SLU. Summary of Product Characteristics Dinalgen 150 mg/ml solution for injection für cattle, pigs and horses. 2022.

[179] Ecuphar Veterinaria SLU. Summary of Product Characteristics Dinalgen 60 mg/ml solution for injection for pigs. 2022.

[180] Ecuphar Veterinaria SLU. Summary of Product Characteristics Dinalgen 300 mg/ml oral solution for cattle and pigs. 2022.

[181] Boehringer Ingelheim Vetmedica GmbH. Summary of Product Characteristics Metacam 15 mg/ml oral suspension for pigs. 2022.

[182] Boehringer Ingelheim Vetmedica GmbH. Summary of Product Characteristics Metacam 5 mg/ml solution for injection for cattle and pigs. 2022.

[183] Boehringer Ingelheim Vetmedica GmbH. Summary of Product Characteristics Metacam 20 mg/ml solution for injection for cattle, pigs and horses. 2022.

[184] Serumwerk Bernburg AG. Summary of Product Characteristics Metapyrin oral 100%, Metapyrin 500 mg/ml Injektionslösung für Pferde, Rinder, Schweine, Hunde. 2020.

[185] Eurovet Animal Health B.V. Solacyl 1000 mg/g, powder for use in drinking water/milk for cattle and pigs. 2021.

[186] SP Veterinaria SA. Tolfedol 40 mg/ml, solution for injection for cattle, pigs, cats and dogs. 2021.

[187] Monteiro B, Lascelles B, Murrell J, Robertson S, Steagall P, Wright B. 2022 WSAVA guidelines for the recognition, assessment and treatment of pain. J Small Anim Pract. 2023;64(4):177–254. 10.1111/jsap.13566.

[188] O’Neill A, Lirk P. Multimodal analgesia. Anesthesiol Clin. 2022;40(3):455–68. 10.1016/j.anclin.2022.04.002. 36049874 10.1016/j.anclin.2022.04.002

[189] Guedes AG, Matthews NS, Hood DM. Effect of ketamine hydrochloride on the analgesic effects of tramadol hydrochloride in horses with signs of chronic laminitis-associated pain. Am J Vet Res. 2012;73(5):610–9. 10.2460/ajvr.73.5.610. 22533391 10.2460/ajvr.73.5.610

[190] Sutherland MA, Davis BL, Brooks TA, McGlone JJ. Physiology and behavior of pigs before and after castration: effects of two topical anesthetics. Animal. 2010;4(12):2071–9. 10.1017/S1751731110001291. 22445382 10.1017/S1751731110001291

[191] Friendship R, Charbonneau G, editors. Pain Control. London Swine Conference; 2013; London, Ontario, Canada: The Pig Site.

[192] Kuhnert L. Die Qual der Wahl des richtigen Analgetikums fürs Schwein. In: Rackwitz R, Truyen U (editors): LBH: 12. Leipziger Tierärztekongress – Tagungsband 3. Leipzig, 2024:40–42.

