# Supplementary material for: Part I: understanding pain in pigs—basic knowledge about pain assessment, measures and therapy
Source: Porcine Health Manag. 2025 Mar 11;11:12. doi: 10.1186/s40813-025-00421-0 (PMC11895375; doi:10.1186/s40813-025-00421-0)
Supplement: Supplementary file 1 — Additional file 1. Metrics of the review. The table provides an overview of the search output and filter processes. [file 40813_2025_421_MOESM1_ESM.docx]

# Supplement 1

## Metrics of the review

| **VetSearch** | **CABI** | |
| --- | --- | --- |
| All n=713 | All n=12 | |
| V1: pain in title n=304 | V1: pain in title: n=0 | |
| V2: pain in subject n= 409 | V2: pain in subject n=12 | |
|  | | |
| duplicates n=275 | duplicates n=0 | |
| false animal n=285 | false animal n=3 | |
| false topic n=51 | false topic n=(0) | |
| access issues n=5 | access issues n=(0) | |
| **appropriate n=97** | **appropriate n=9** | |
|  |  | |
| Screening I : 55 | |  |
| Screening II: 49 | |  |
