# Supplementary figures and images for: Part I: understanding pain in pigs—basic knowledge about pain assessment, measures and therapy
Source: Porcine Health Manag. 2025 Mar 11;11:12. doi: 10.1186/s40813-025-00421-0 (PMC11895375; doi:10.1186/s40813-025-00421-0)

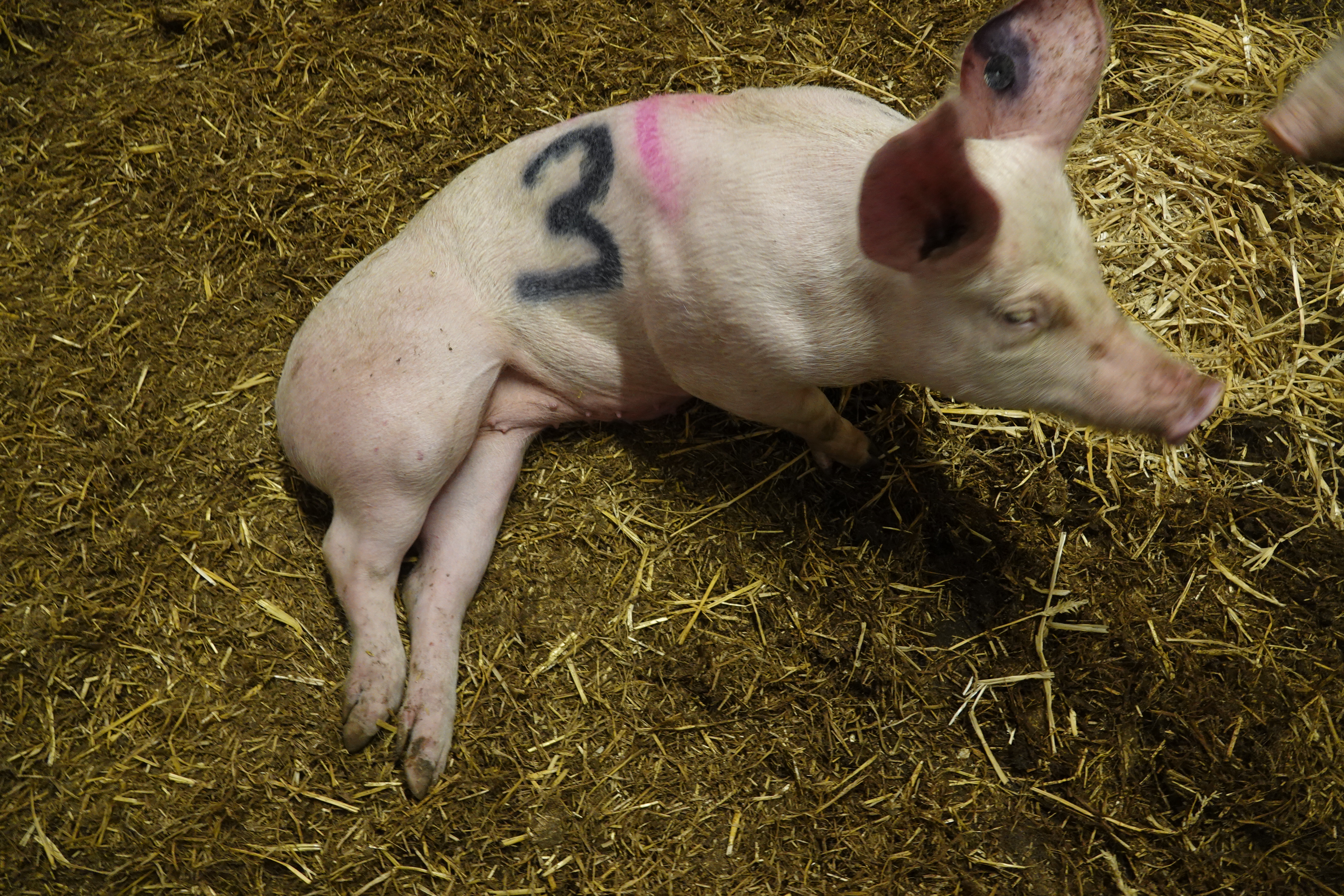

Supplement: Supplementary file 8 — Additional file 8: Bending the spine. The picture shows a pig unburden a hind leg by bending the spine. Permission to reuse the materials for the purpose of illustrating the signs and arguments of the authors in this article is granted. [file 40813_2025_421_MOESM8_ESM.jpg]
